# Using host traits to predict reservoir host species of rabies virus

**DOI:** 10.1371/journal.pntd.0008940

**Published:** 2020-12-08

**Authors:** Katherine E. L. Worsley-Tonks, Luis E. Escobar, Roman Biek, Mariana Castaneda-Guzman, Meggan E. Craft, Daniel G. Streicker, Lauren A. White, Nicholas M. Fountain-Jones

**Affiliations:** 1 Department of Veterinary Population Medicine, University of Minnesota, Saint Paul, Minnesota, United States of America; 2 Department of Fish and Wildlife Conservation, Virginia Tech, Blacksburg, Virginia, United States of America; 3 Institute of Biodiversity, Animal Health and Comparative Medicine, University of Glasgow, Glasgow, United Kingdom; 4 Department of Ecology, Evolution and Behavior, University of Minnesota, Saint Paul, Minnesota, United States of America; 5 MRC-University of Glasgow Centre for Virus Research, Glasgow, United Kingdom; 6 National Socio-Environmental Synthesis Center, University of Maryland, Annapolis, Maryland, United States of America; 7 School of Natural Sciences, University of Tasmania, Hobart, Australia; Universitetet i Oslo, NORWAY

## Abstract

Wildlife are important reservoirs for many pathogens, yet the role that different species play in pathogen maintenance frequently remains unknown. This is the case for rabies, a viral disease of mammals. While Carnivora (carnivores) and Chiroptera (bats) are the canonical mammalian orders known to be responsible for the maintenance and onward transmission of rabies *Lyssavirus* (RABV), the role of most species within these orders remains unknown and is continually changing as a result of contemporary host shifting. We combined a trait-based analytical approach with gradient boosting machine learning models to identify physiological and ecological host features associated with being a reservoir for RABV. We then used a cooperative game theory approach to determine species-specific traits associated with known RABV reservoirs. Being a carnivore reservoir for RABV was associated with phylogenetic similarity to known RABV reservoirs, along with other traits such as having larger litters and earlier sexual maturity. For bats, location in the Americas and geographic range were the most important predictors of RABV reservoir status, along with having a large litter. Our models identified 44 carnivore and 34 bat species that are currently not recognized as RABV reservoirs, but that have trait profiles suggesting their capacity to be or become reservoirs. Further, our findings suggest that potential reservoir species among bats and carnivores occur both within and outside of areas with current RABV circulation. These results show the ability of a trait-based approach to detect potential reservoirs of infection and could inform rabies control programs and surveillance efforts by identifying the types of species and traits that facilitate RABV maintenance and transmission.

## Author summary

Rabies, a fatal viral disease transmitted via the bite of an infected animal, causes over 59,000 human deaths each year. While domestic dogs are responsible for most human cases, wild animals, particularly carnivores and bats, play an essential role in rabies persistence. Several carnivore and bat species have been identified in the field and laboratory as important rabies reservoirs that can maintain different rabies variants. However, the role of many other carnivore and bat species remains unknown and additional insights are hampered by logistical and funding limitations of field and laboratory research. Using machine learning, we used information on known rabies reservoirs to forecast previously unidentified rabies reservoirs. Our models predicted 44 carnivore and 34 bat species that could already be serving as unrecognized rabies reservoirs or that have trait profiles suggesting their capacity to become reservoirs via future host shifting. In many cases, the distribution of these predicted reservoirs overlaps geographically with known reservoir ranges. Our findings can help understand rabies circulation in wildlife and is a first step to guide future epidemiological surveillance in neglected species.

## Introduction

Most wildlife pathogens can infect multiple host species. However, typically only a few host species act as reservoirs, i.e., are responsible for maintaining a pathogen in a region in the long term and for transmitting it to other species of concern [1,2]. This is because most host species lack intrinsic competency to contribute to transmission [1–3]. The likelihood of host species to be reservoirs will depend on both their characteristics and the life cycle and infection biology of the pathogen, such that some host traits may favor maintenance of some pathogens, but not others. Determining whether host species have the characteristics to maintain certain pathogens can be extremely difficult to quantify in the field and often requires performing in-depth investigations. Thus, only a limited number of wildlife species have been examined as potential reservoir candidates (e.g., [4]) and the focus has been on those that overlap with people and domestic animals the most [5,6].

Neglecting the role of unrecognized reservoir species present in a community may have negative consequences for disease prevention and control [2]. For example, foot-and-mouth disease (FMD) in South Africa was previously perceived as circulating solely in African buffalo (*Syncerus caffer*) and livestock, but empirical evidence revealed that impala (*Aepyceros melampus*) may play a critical role for propagating FMD [7]. Further, given the current and future shifts in climatic and environmental conditions [8], wildlife community assemblages are expected to change [9,10]. Therefore, the role of species in reservoir communities is also likely to shift. This means that while a host species may not currently play a role in the transmission and persistence of a pathogen, its reservoir status may change in the future. Hence, there is a pressing need to develop approaches that can rapidly identify potential reservoir species, without necessarily having to perform in-depth, long-term field investigations.

One promising approach for discovering unknown reservoirs is to identify characteristics that ‘known’ reservoirs of a particular pathogen or pathogens have in common and use these traits to quantify the likelihood that other understudied species could act as reservoirs. This trait-based approach has only recently been used for understanding the ecology of infectious diseases in wildlife and plants (e.g., [11–14]) but has already identified some interesting patterns. For example, two traits that appear to emerge as important in different host-pathogen systems are animal birth rate and longevity (e.g., [11,15–17] but see [18]). Animals that tend to have a high birth rate and/or a short life-span are predicted to be reservoirs for many types of pathogens (e.g., *Borrelia burgdorferi* and flaviviruses [19,20]). Thus, traits identified in typically well studied, accessible species can be applied to less well-studied species, directing research and surveillance effort.

Here, we used a trait-based approach to identify candidate wildlife species potentially involved in the transmission and maintenance of rabies *Lyssavirus* (RABV). RABV continues to be a major public health concern as it is responsible for over 59,000 deaths each year [21], with economic costs estimated to be as high as $6 billion annually [22]. People generally become infected with RABV via the bite of an infected animal. While all mammal species can become infected with RABV, relatively few carnivore and bat species appear to act as reservoirs and sustain transmission independently [23,24]. In many developing countries, particularly African and Asian countries, the domestic dog (*Canis lupus familiaris*) is considered a primary reservoir [25–27]. While local wild carnivores can, in some cases, contribute to the maintenance of certain RABV variants [28–30], the role of most wildlife species remains relatively uncharacterized because of the overwhelming number of canine cases and lack of routine wildlife surveillance systems or diagnostic tests [31]. In countries with effective dog vaccination, domestic dogs no longer play a role in the maintenance of RABV [21,32]. However, RABV persists in many of these countries due to wildlife species that maintain independent RABV lineages [33,34]. For example, in the United States, over the past four decades, 90% of reported rabies cases have been from wildlife [35,36]. While key carnivore and bat species have been recognised as primary reservoirs [37], novel reservoirs for RABV are predicted to emerge due to recurring cross-species transmission and/or sustained transmission events (e.g., [38,39]). Thus, anticipating future spillover events is vital if we are to ensure current control programs continue to be successful.

We applied machine learning to life history and ecological data we compiled for both known and previously unidentified RABV reservoirs to (i) identify traits associated with being a reservoir for RABV; (ii) predict which species could be unrecognized or future reservoirs; (iii) determine the contribution of each specific trait to predicted reservoir status; and (iv) investigate the geographic distribution of known and predicted RABV reservoirs to identify hotspots of historic and potential RABV spillover and host shifts. While all mammals are generally susceptible to RABV, we focused on those within the orders Carnivora and Chiroptera because of their established role in the maintenance and onward transmission of RABV [40–42]. Further, since RABV does not circulate in bats outside of the Americas [43,44], we focused on bat species occurring in the Americas.

## Methods

### Reservoir assignment and data collection

To determine the reservoir status of each carnivore and bat species, we conducted a general review of the literature. The literature review was performed in 2017 and articles were collected from Google Scholar using the keywords: ‘rabies’ AND ‘reservoir’, followed by each species’ scientific name. If the keyword ‘rabies’ and the species scientific name appeared in articles, articles were read in full. Species were classified as reservoirs only if they fell under one of two definitions: a conservative and a liberal definition. The conservative definition labelled species as reservoirs for RABV if they were described as ‘reservoirs’ in the article and were associated with one or several genetically distinct virus variants [45]. The liberal definition labelled species as reservoirs if individuals of the species had been recorded as infected or had antibodies against RABV, and had been suggested in the article to play a role in RABV transmission (e.g., described as being ‘a primary host’). We classified the species into the conservative or liberal group if this was supported by at least one article. Species outside these two groups were classified as not having enough evidence for being a reservoir for RABV. Reservoir assignment data are available at our online data repository ‘Predicting-rabies-reservoirs’ (https://github.com/worsl001/Predicting-rabies-reservoirs). Reservoir assignment data are also available at ‘ReservoirFinder’ (https://github.com/whit1951/ResevoirFinder), where wildlife reservoir classification of other multi-host pathogens can be deposited (e.g., *Leptospira*, *Hantavirus*, *Leishmania*).

### Species traits

The majority of species traits were obtained from the PanTHERIA database [46]. Of the 45 PanTHERIA traits, 15 were examined for carnivores and 9 for bats. The other traits were excluded either because more than 50% of species had missing values, traits had no hypothesized or plausible link to RABV reservoirs (e.g., mean monthly evapotranspiration rate), traits were highly correlated with other traits (i.e., *ρ* > 0.7; e.g., diet breadth and trophic level), or traits presented little to no variation (e.g., for bats, 97% of species had the same habitat breadth value). For bats, since the litter size trait was relatively uniform across species (median: 0.99, range: 0.98–3.12), it was reclassified into a binary variable (zero for litter size ≤1 and one for litter size >1). For carnivores, we included two additional traits gathered from the Animal Diversity Web (https://animaldiversity.org/): sociality and mono/polygamous. We also included information on species phylogenetic grouping based on well resolved phylogenies for each group (carnivores; [47], bats; [48]), to account for the statistical non-independence of species due to common ancestry [49]. We calculated the patristic distance (i.e., the sum of branch lengths between two tips) for each group and then applied Principal Coordinate Analysis (PCoA) to reduce the dimensions of each respective matrix. The first PCoA quantified the broadest variation across the phylogeny (e.g., suborder variation) with subsequent axes capturing progressively smaller amounts of phylogenetic variation (e.g., S1 Fig). We included the top three or four principal coordinate eigenvalues as traits (for carnivores, we excluded the fourth principal coordinate because of it being highly correlated with age at sexual maturity). Nine carnivore and eight bat species were excluded because of having no trait data in the PanTHERIA database (carnivores: *Genetta bourloni*, *Genetta poensis*, *Crossarchus platycephalus*, *Meles anakuma*, *Meles leucurus*, *Neovison macrodon*, *Spilogale angustifrons*, *Zalophus japonicus*, and *Zalophus wollebaeki*; bats: *Carollia sowelli*, *Histiotus humboldti*, *Lasiurus atratus*, *Lasiurus salinae*, *Lasiurus varius*, *Mormoops magna*, *Nycticeius aenobarbus*, and *Phyllonycteris major*). None of the 17 excluded carnivore and bat species are known to be reservoirs for RABV based on the literature.

### Identifying traits predictive of reservoir status

To identify traits that best predict the reservoir status of each species, we used gradient boosting machine (GBM) models in the statistical program R (version 4.0.2) [50] using the ‘*caret*’ and ‘*gbm*’ packages (version 6.0–86 and 2.1.8, respectively) [51,52]. We chose to use GBM models over more traditional regression techniques as GBM models offer a flexible and powerful classification approach that can model nonlinear effects and interactions and provide high predictive performance without overfitting [53,54]. Further, GBM models can efficiently analyze a large number of predictors, including categorical predictors, whilst accounting for missing data [54]. We followed the analytical framework proposed by Fountain-Jones et al. [55].

We ran two models for each mammal order (i.e., Carnivora and Chiroptera): 1) a conservative model using the conservative definition of a reservoir species for RABV; and 2) a liberal model using the liberal definition of a reservoir species for RABV. For the carnivore GBM models, we used five categorical and 15 continuous predictor variables (i.e., traits), and for the bat models we used two categorical and 11 continuous variables. For each model, species were split into two groups: a training set (80%) and a testing set (20%). Models were trained using 10-fold cross-validation of the training set. Since we had substantially fewer reservoirs than non-reservoirs in each dataset, we performed down-sampling, which randomly subsets the classes in the training model to avoid potential class imbalance as described elsewhere [55]. Cross-validation was used to determine model accuracy, sensitivity, and specificity based on a confusion matrix. Accuracy represents the proportion of species that were correctly classified as reservoirs or non-reservoirs, sensitivity represents the proportion of species that were correctly classified as reservoirs, and specificity represents the proportion of species that were correctly classified as non-reservoirs. The test set was used to explore model performance on a set of observations not included in model construction. To find the optimal combination of tuning parameters suitable for each GBM model, we used ‘expand.grid’ in the ‘*caret’* package, which optimizes the learning rate, number of classification trees, and shrinkage [56].

After model training, we quantified variable importance based on all observations using the ‘*iml*’ package (version 0.10.0) [57]. Variables are considered to be ‘important’ if model error increases after permutation [58]. The effect of each variable on the response was visualized by creating partial dependence plots using the ‘*pdp*’ package (version 0.7.0) [59]. To visualize how the predicted probability of being a reservoir for RABV varied by species, we included individual conditional expectation (ICE) curves in each partial dependence plot [60].

### Reservoir prediction and trait importance

To identify candidate reservoirs for RABV and determine how each trait contributed to the predicted reservoir status of each species, we used a cooperative game theory approach—the Shapley value [61], using ‘*iml*’ [57]. The Shapley value aims to explain the prediction of the GBM model for each observation (in this case a host species). Hence, for each species, the Shapley value uses information from the GBM model to assess the contribution of each trait on the models’ prediction (i.e., being or not a reservoir for RABV). Positive Shapley values indicate that predictors are increasing the likelihood that the outcome is positive (i.e., a species is a reservoir for RABV), and negative Shapley values indicate that predictors are increasing the likelihood that the outcome is negative (i.e., a species is not a reservoir for RABV). Importantly, the Shapley value uses a different criterion for classifying reservoirs than the GBM model alone. GBM predictions are based on a 0.5 probability (above 0.5 species are considered reservoirs, below 0.5 species are considered non-reservoirs). Shapley values are based on the difference between the GBM predicted value for the species of interest and the average GBM predicted value for all species. Thus, the Shapley value classification criterion is arguably more insightful than the GBM because it evaluates the role of a reservoir species in the context of all other species. Additionally, the Shapley value not only indicates whether species are incorrectly classified (by combining the Shapley values of all predictors) but also provides insight into the importance of each predictor at influencing the reservoir outcome for each species. Thus, a species is classified as a reservoir for RABV if the Shapley scores of predictors sum to a value that is > 0. Otherwise, the species is either classified as not being a reservoir for RABV (if the Shapley scores sum to a value < 0) or as unknown (if the Shapley scores sum to a value that is equal to 0).

### Mapping the geographic distribution of known and predicted RABV reservoirs

The geographic range of known and predicted reservoirs for RABV were collected from the International Union for Conservation of Nature’s (IUCN) Red List database (www.iucnredlist.org). Pixel values of species ranges were reclassified to be binary (i.e., a pixel value of 1 indicates the species is present and a pixel value of 0 indicates the species is absent). The ranges of species belonging to the same reservoir group (e.g., known carnivore reservoirs based on the conservative criteria) were stacked using the ‘*rgdal*’ package (version 1.4–8) [62]. Maps were created using the ‘*rasterVis*’ package (version 0.47) [63] to identify areas where predicted reservoir species are likely to co-occur.

## Results

### Carnivores

#### Traits associated with being a reservoir for RABV

**Fig 1:**
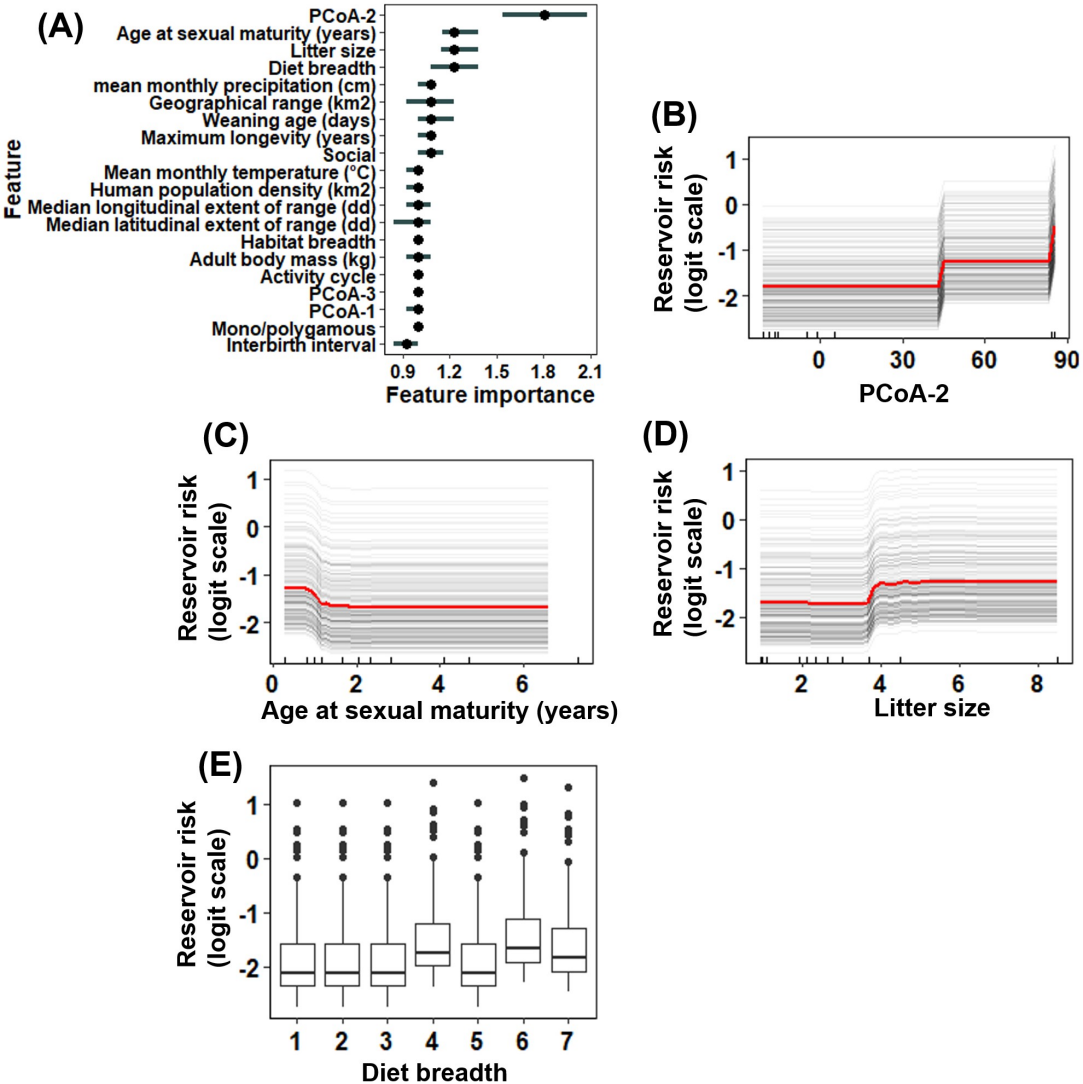
Traits predictive of carnivore RABV reservoir status based on the conservative criteria. **(A)** Trait importance (measured based on model error change after permutation) and **(B)-(E)** partial dependence plots relating RABV reservoir status (the log-odds scale of being a reservoir for RABV) with the four most predictive carnivore traits based on the conservative criteria. In panel **(A)**, PCoA1-3 refers to the first to third principal coordinates of species phylogenetic relatedness. In panels **(B)-(D)**, the red line represents the mean prediction across all species. The grey lines are the Individual Conditional Expectation (ICE) curves, which illustrate the predictive change in each species being a reservoir for RABV as each feature changes. The tick marks along the x-axis represent the deciles of each trait value included in model training. The median age at sexual maturity was ~2 years and the median litter size was 2.35.

Of the 277 carnivore species for which sufficient data were available, 23 (8.3%) were identified as being reservoirs for RABV based on the conservative criteria, and 27 (9.7%) based on the liberal criteria. The conservative and liberal models had an accuracy of 67.16% (sensitivity = 75.79%) and 65.89% (sensitivity = 70.0%), respectively (S1 Table). For the conservative model, species phylogenetic grouping (inferred from the second principal coordinate (PCoA-2)) was the most important predictor of RABV reservoir status (prediction error increased by 1.81 orders of magnitude after permutation; Fig 1A). Next most important were age at sexual maturity, median litter size, and diet breadth (error increased by 1.23 orders of magnitude after permutation for all three traits; Fig 1A). Carnivore species were more likely to be reservoirs for RABV if they were part of the Canidae family (PCoA-2 values ranging from 83–86) (Fig 1B; S2 Fig). More generally, the likelihood for carnivores to be RABV reservoirs decreased with age at sexual maturity (Fig 1C) but increased as the number of young per litter increased (Fig 1D) and as the number of dietary categories increased (Fig 1E). All top traits identified in the conservative model were also identified as the top traits in the liberal model (S3 Fig).

#### Predicted RABV reservoirs

**Fig 2:**
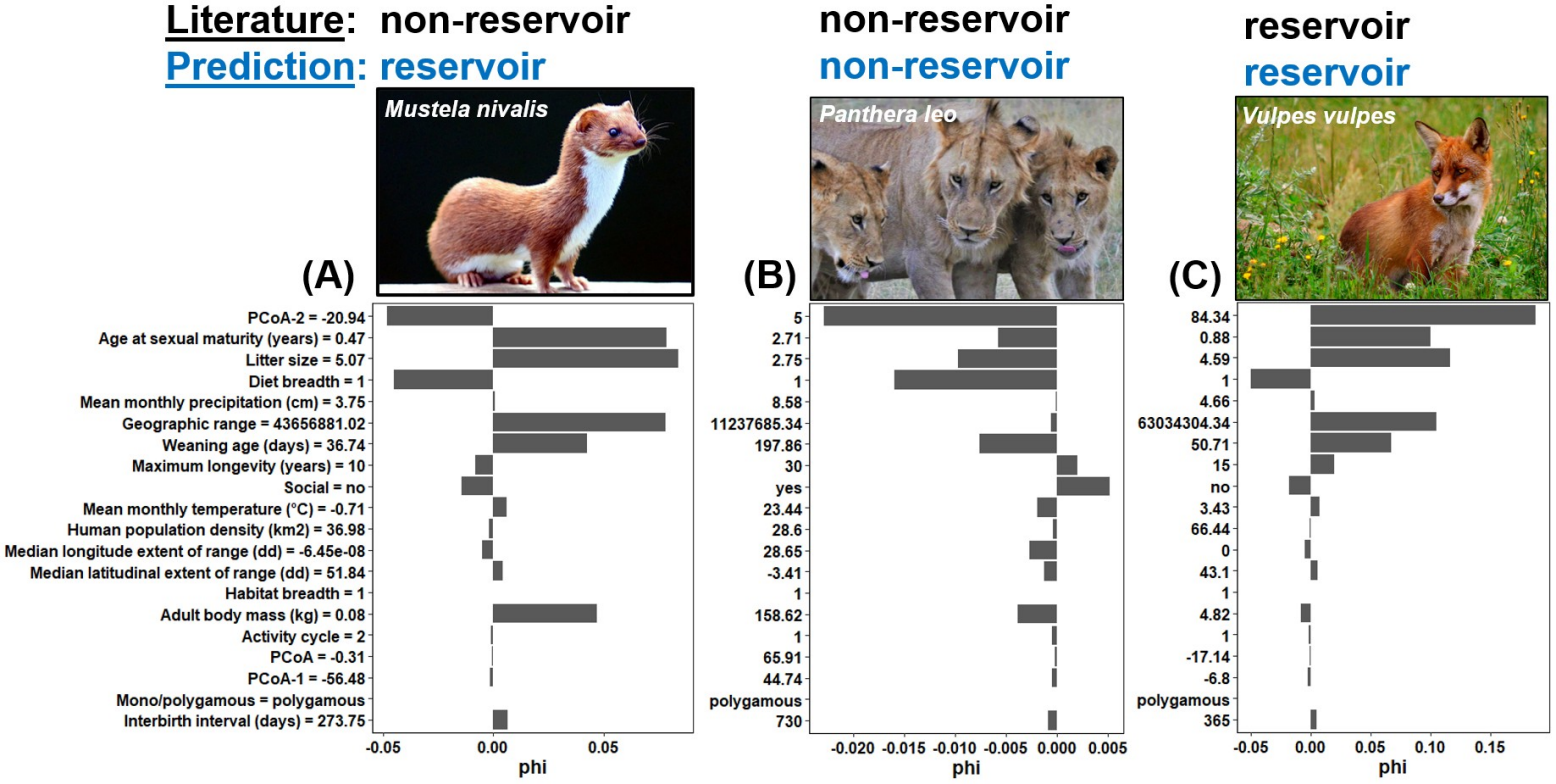
Illustrative examples of (A) a predicted reservoir: The least weasel (*Mustela nivalis*); (B) a non-reservoir: The lion (*Panthera leo*); and (C) a known reservoir: The red fox (*Vulpes vulpes*). Bars denote trait importance based on the Shapley value (phi). Positive Shapley values indicate that predictors are increasing the likelihood that the outcome is positive (i.e., the likelihood a species is a reservoir for RABV), and negative Shapley values indicate that predictors are increasing the likelihood that the outcome is negative (i.e., the likelihood a species is not a reservoir for RABV). Values next to each trait represent the trait measure for each one of the three species (e.g., obtained from the PanTHERIA database). Least weasel and red fox photos were obtained from Wikimedia Commons (https://commons.wikimedia.org/) and the lion photo was the authors’ contribution. The text at the top of each image represents the reservoir status of each species based on the literature (black) and GBM models (blue).

The model predicted 38 carnivore species that could act as reservoirs for RABV in the conservative model (Table 1) and 39 in the liberal model (S2 Table) (summing to a total of 44 species across the two models). Further, the conservative model predicted three currently recognized carnivore reservoirs for RABV to be non-reservoirs: the Chinese ferret-badger (*Melogale moschata*), the kinkajou (*Potos flavus*), and the raccoon (*Procyon lotor*). The liberal model predicted two currently recognized carnivore reservoirs to be non-reservoirs: the meerkat (*Suricata suricatta*) and the spotted hyena (*Crocuta crocuta*). In the conservative model, several species of the Canidae, Herpestidae, and Mustelidae families were predicted to be reservoirs for RABV (e.g., the culpeo (*Lycalopex culpaeus*), the common kusimanse (*Crossarchus obscurus*), and the least weasel (*Mustela nivalis*); Table 1). For species from the Mustelidae family, this was partly because individuals from these species tend to reproduce at a young age and have large litters (e.g., the least weasel; Fig 2A). Non-canids that do not reproduce at a young age, have small litters (less than ~3.5 young per litter), and have only one dietary category were less likely to be reservoirs for RABV (e.g. the lion (*Panthera leo*; Fig 2B). In contrast, an empirically-recognized reservoir for RABV, like the red fox (*Vulpes vulpes*), was classified as being a reservoir in our game theory model partly because of individuals reproducing at a young age (~11 months) and having large litters (4–5 young per litter) (Fig 2C).

**Table 1:** Carnivore species predicted to be reservoirs for RABV based on the conservative criteria. Since there is inherent variation when performing permutations, species with Shapley values close to zero (especially those < 0.1) should be considered with caution.

Species	Family	Shapley value
Culpeo (*Lycalopex culpaeus*)	Canidae	0.42
Swift fox (*Vulpes velox*)	Canidae	0.34
Common kusimanse (*Crossarchus obscurus*)	Herpestidae	0.23
Dhole (*Cuon alpinus*)	Canidae	0.22
Least weasel (*Mustela nivalis*)	Mustelidae	0.22
Fennec fox (*Vulpes zerda*)	Canidae	0.21
African wild dog (*Lycaon pictus*)	Canidae	0.2
Marsh mongoose (*Atilax paludinosus*)	Herpestidae	0.19
Bush dog (*Speothos venaticus*)	Canidae	0.19
Pampas fox (*Lycalopex gymnocercus*)	Canidae	0.17
Long-tailed weasel (*Mustela frenata*)	Mustelidae	0.17
Steppe polecat (*Mustela eversmanii*)	Mustelidae	0.16
Cape fox (*Vulpes chama*)	Canidae	0.16
Stoat (*Mustela erminea*)	Mustelidae	0.13
Ring-tailed cat (*Bassariscus astutus*)	Procyonidae	0.12
Blanford’s fox (*Vulpes cana*)	Canidae	0.12
Tibetan fox (*Vulpes ferrilata*)	Canidae	0.11
Kit fox (*Vulpes macrotis*)	Canidae	0.1
Maned wolf (*Chrysocyon brachyurus*)	Canidae	0.1
Island fox (*Urocyon littoralis*)	Canidae	0.1
Indian grey mongoose (*Herpestes edwardsi*)	Herpestidae	0.08
Banded mongoose (*Mungos mungo*)	Herpestidae	0.07
Pale fox (*Vulpes pallida*)	Canidae	0.07
European polecat (*Mustela putorius*)	Mustelidae	0.06
Meerkat (*Suricata suricatta*)	Herpestidae	0.05
Pygmy spotted skunk (*Spilogale pygmaea*)	Mephitidae	0.05
Striped polecat (*Ictonyx striatus*)	Mustelidae	0.04
European mink (*Mustela lutreola*)	Mustelidae	0.04
African civet (*Civettictis civetta*)	Viverridae	0.03
Honey badger (*Mellivora capensis*)	Mustelidae	0.02
Ethiopian wolf (*Canis simensis*)	Canidae	0.01
South American grey fox (*Lycalopex griseus*)	Canidae	0.01
Bengal fox (*Vulpes bengalensis*)	Canidae	0.01
Striped hyena (*Hyaena hyaena*)	Hyaenidae	0.01
Short-eared dog (*Atelocynus microtis*)	Canidae	0.01
American hog-nosed skunk (*Conepatus leuconotus*)	Mephitidae	0.01
Sable (*Martes zibellina*)	Mustelidae	0.01
Brown bear (*Ursus arctos*)	Ursidae	0.01

#### Geographic range of known and predicted reservoirs for RABV

**Fig 3:**
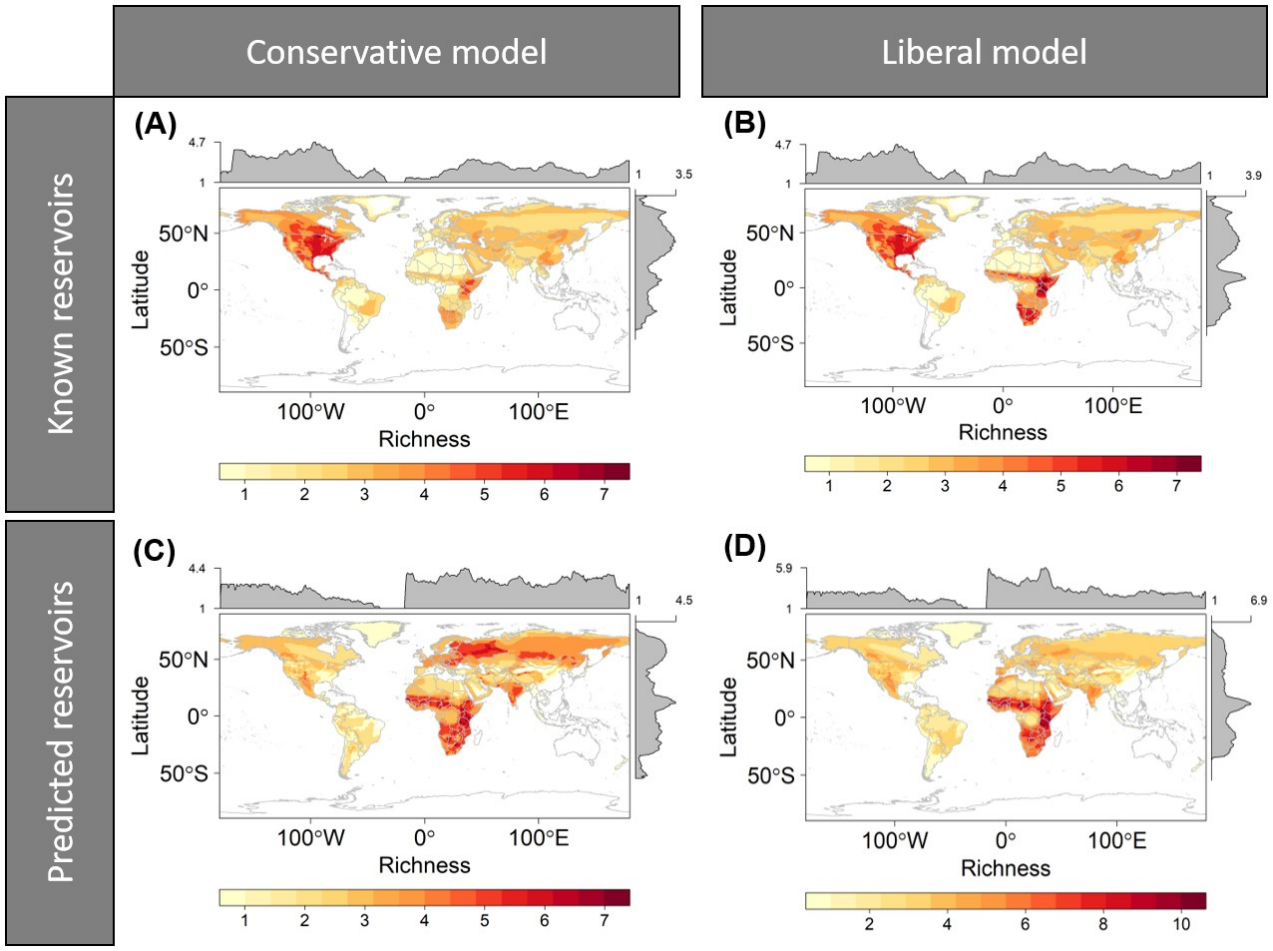
Distribution of carnivore RABV reservoirs identified by the conservative model ((A) and (C)) and the liberal model ((B) and (D)). Panel **(A)** and **(B)** represent known reservoirs, and panel **(C)** and **(D)** predicted reservoirs. The maps show areas with high (red), moderate (orange), and low (yellow) number of carnivore reservoirs for RABV. Grey histograms represent the richness level (i.e., the number of reservoir species in each pixel).

The greatest richness of known carnivore RABV reservoirs (~5–7 species) mostly clustered in North America, parts of Mexico and Central America, and East Africa for the conservative model (Fig 3A), along with central and southern Africa in the liberal model (Fig 3B). Predicted carnivore reservoirs based on the conservative model mostly clustered in southern central US, southern, central, and eastern Africa, as well as parts of south-eastern Europe, western and southern Russia, and eastern India (Fig 3C). Predicted reservoirs based on the liberal model clustered for the most part in southern, central, and eastern Africa (Fig 3D).

### Chiroptera

#### Traits associated with being a reservoir for RABV

**Fig 4:**
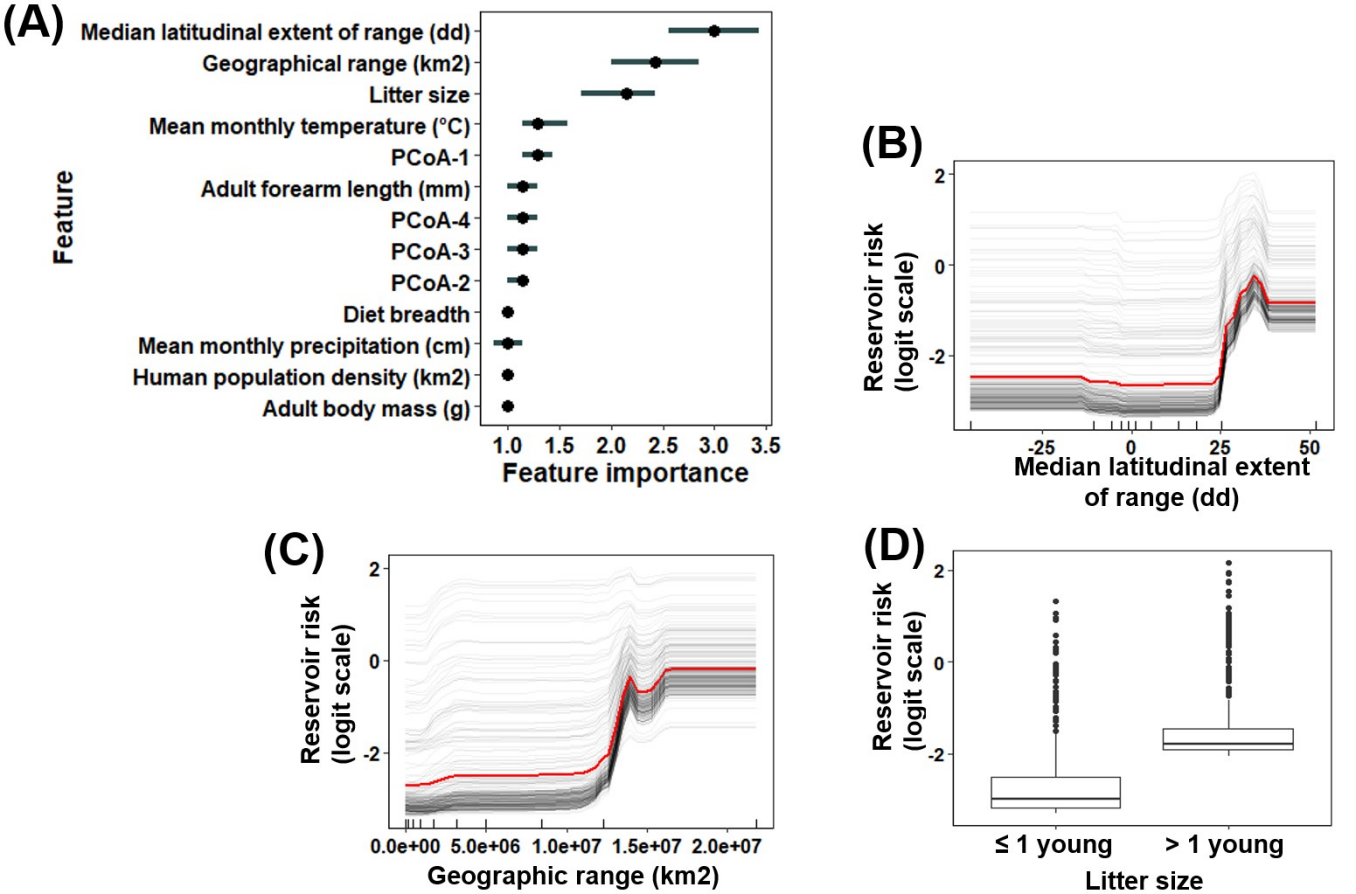
Traits predictive of bat RABV reservoir status based on the conservative criteria. **(A)** Trait importance (measured based on model error change after permutation) and **(B)-(D)** partial dependence plots relating reservoir status (the log-odds scale of being a reservoir for RABV) with the three most predictive bat traits based on the conservative criteria. In panel **(A)**, PCoA1-4 refers to the first to fourth principal coordinates of species phylogenetic relatedness. In panels **(B)-(C)**, the red line represents the mean prediction across all species. The grey lines are the Individual Conditional Expectation (ICE) curves, which illustrate the predictive change in each species being a reservoir for RABV as each feature changes. The tick marks along the x-axis represent the deciles of each trait values included in model training.

Of the 326 bat species for which sufficient data were available, 29 (8.9%) were identified as being reservoirs for RABV based on the conservative criteria, and 41 (12.6%) based on the liberal criteria. The conservative and liberal models had an accuracy of 82.59 (sensitivity = 83.75%) and 82.41 (sensitivity = 87.58%), respectively (S1 Table). For the conservative model, median latitudinal extent of range was the most important predictor of RABV reservoir status, followed by geographic range (km^2^), and litter size, (prediction error increased by 3, 2.43, 2.14 orders of magnitude after permutation, respectively; Fig 4A). Bat species that resided in North America, ranged over 1 x 10^7^ km^2^, and had more than one young per litter were more likely to have been predicted as reservoirs for RABV (Fig 4B–4D). All top traits identified in the conservative model were also identified as the top traits in the liberal model (S4 Fig).

#### Predicted RABV reservoirs

**Fig 5:**
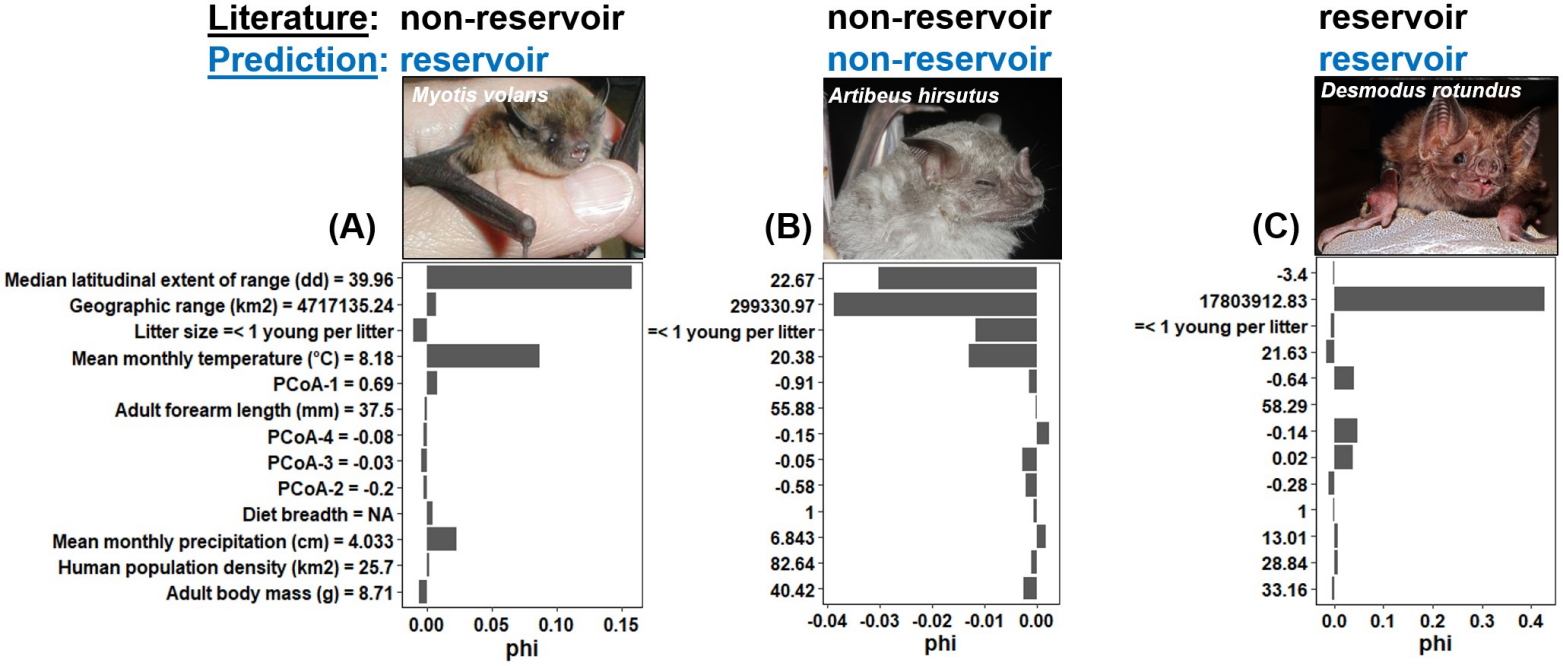
Illustrative examples of (A) a predictive reservoir: The long-legged myotis (*Myotis volans*); (B) a non-reservoir: The hairy fruit-eating bat (*Artibeus hirsutus*); and (C) a known reservoir: The vampire bat (*Desmodus rotundus*). Bars denote trait importance based on the Shapley value (phi). Positive Shapley values indicate that predictors are increasing the likelihood that the outcome is positive (i.e., a species is a reservoir for RABV), and negative Shapley values indicate that predictors are increasing the likelihood that the outcome is negative (i.e., a species is not a reservoir for RABV). Values next to each trait represent the trait measure for each one of the three species (obtained from the PanTHERIA database). All photos were obtained from Wikimedia Commons (https://commons.wikimedia.org/). The text at the top of each image represents the reservoir status of each species based on the literature (black) and GBM models (blue).

The conservative model predicted 16 bat species that could act as reservoirs for RABV (Table 2) and the liberal model predicted 34 (S3 Table) (summing to a total of 34 species across the two models). All recognized RABV reservoirs were correctly classified as reservoirs in both conservative and liberal models, except for two in the conservative model: the black myotis (*Myotis nigricans*) and the little brown bat (*Myotis lucifugus*); and four in the liberal model: the dark fruit-eating bat (*Artibeus obscurus*), the little brown bat (*Myotis lucifugus*), the tropical big-eared brown bat (*Histiotus velatus*), and the western yellow bat (*Lasiurus xanthinus*). In the conservative model, of the newly identified reservoirs, the long-legged myotis (*Myotis volans*) was predicted to be a reservoir in part because it occurs in North America (Fig 5A). The hairy fruit-eating bat (*Artibeus hirsutus*), which ranges over a relatively small area in Mexico, was less likely to be a reservoir for RABV (Fig 5B). A well-recognized reservoir for RABV, the vampire bat (*Desmodus rotundus*), was predicted to be a reservoir because of having a large geographic range (Fig 5C).

**Table 2:** Bat species predicted to be RABV reservoirs based on the conservative criteria. Since there is inherent variation when performing permutations, species with Shapley values close to zero (especially those < 0.1) should be considered with caution.

Species	Family	Shapley value
Spotted bat (*Euderma maculatum*)	Vespertilionidae	0.47
Southern yellow bat (*Lasiurus ega*)	Vespertilionidae	0.34
Long-legged myotis (*Myotis volans*)	Vespertilionidae	0.26
Eastern small-footed myotis (*Myotis leibii*)	Vespertilionidae	0.21
Dark-nosed small-footed myotis (*Myotis melanorhinus*)	Vespertilionidae	0.18
Western mastiff bat (*Eumops perotis*)	Molossidae	0.15
Gray bat (*Myotis grisescens*)	Vespertilionidae	0.1
Northern long-eared myotis (*Myotis septentrionalis*)	Vespertilionidae	0.09
Pallas’s long-tongued bat (*Glossophaga soricina*)	Phyllostomidae	0.08
Big free-tailed bat (*Nyctinomops macrotis*)	Molossidae	0.07
Indiana bat (*Myotis sodalis*)	Vespertilionidae	0.05
Dwarf bonneted bat (*Eumops bonariensis*)	Molossidae	0.38
Armenian whiskered bat (*Myotis hajastanicus*)	Vespertilionidae	0.03
Rafinesque’s big-eared bat (*Corynorhinus rafinesquii*)	Vespertilionidae	0.02
Little yellow-shouldered bat (*Sturnira lilium*)	Phyllostomidae	0.02
Jamaican fruit bat (*Artibeus jamaicensis*)	Phyllostomidae	0.02

#### Geographic range of known and predicted RABV reservoirs

**Fig 6:**
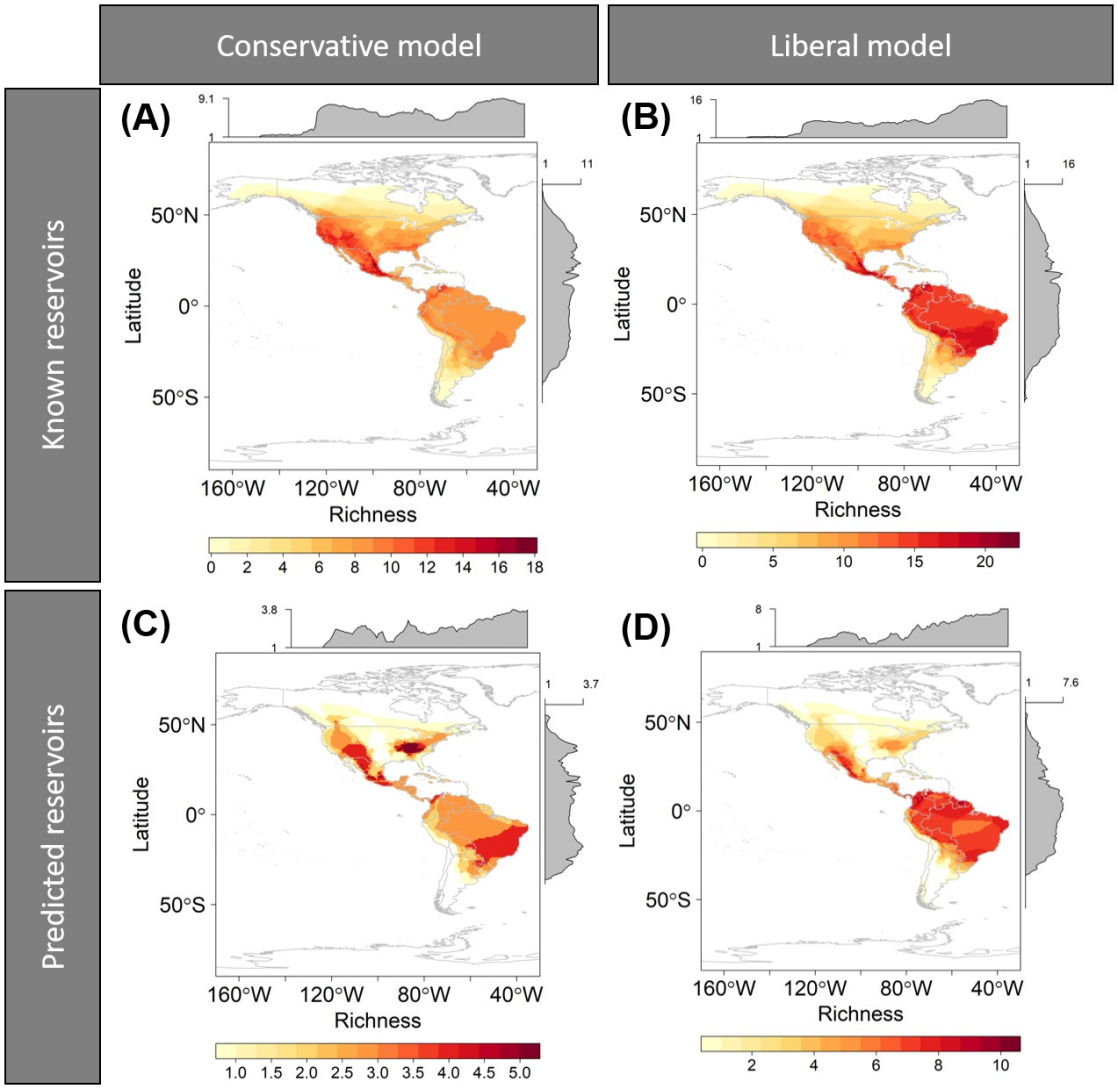
Geographic distribution of bat RABV reservoirs identified by the conservative model ((A) and (C)) and the liberal model ((B) and (D)). Panel **(A)** and **(B)** represent known reservoirs, and panel **(C)** and **(D)** predicted reservoirs. The maps show areas with high (red), moderate (orange), and low (yellow) number of bat RABV reservoirs. Grey histograms represent the richness level (i.e., the number of reservoir species in each pixel).

The greatest richness of known bat RABV reservoirs (~14–18 species) clustered in Mexico and south-western parts of the US for the conservative model (Fig 6A), along with parts of Central America and northern South America in the liberal model (~15–20 species; Fig 6B). The greatest richness of predicted reservoirs based on the conservative model (~4–5 species) clustered mostly in Mexico, south-eastern and western US, southern Brazil, and northern Colombia (Fig 6C), and clustered in western Mexico and northern South America based on the liberal model (~7–10 species) (Fig 6D).

## Discussion

Up to 68 carnivore and bat species across the globe are known to be RABV reservoirs according to our definition, and our models predicted there to be an additional 78 potential reservoir species. The traits that emerged as most important for predicting RABV reservoir status for carnivores were phylogenetic grouping, litter size, and age at sexual maturity. For bats, position along the latitudinal gradient of the Americas, geographic range, along with litter size were the most important traits. Interestingly, while the top traits identified by the GBM models were important at predicting the reservoir status of carnivore and bat species, the contribution of each trait varied by species within each order. Additionally, mapping the spatial distribution of known and predicted reservoirs for RABV revealed that predicted carnivore and bat reservoirs both occurred within the range of known RABV reservoirs and beyond. This suggests that some reservoir species might be missed in known RABV hotspots, that several species could be facilitating or have the potential to facilitate RABV maintenance outside of these areas, and that predicted reservoir species could become RABV reservoirs if the right strain was introduced.

Age at sexual maturity and having large litters were among the most important traits for being a carnivore RABV reservoir, in both the conservative and liberal models. These two traits are associated with species having short lifespans and reproducing rapidly, and have been identified as important for predicting wildlife reservoir status for other pathogens [16,64]. These types of traits may also be important for determining the maintenance success of pathogens for which density-dependent transmission has been hypothesised, such as RABV ([65] although see [66,67]). Thus, carnivore reservoirs for RABV appear to have similar characteristics as reservoirs of other directly transmitted pathogens in that they tend to have faster life history characteristics than non-reservoir species. While several other life-history characteristics appeared to play a less important role in influencing the reservoir status of carnivore species, the finding that most predicted carnivore RABV reservoirs tended to be members of the Canidae, Herpestidae, and Mustelidae families suggests that other traits specific to these families are likely to be important.

It is noteworthy that few carnivore species were predicted to be RABV reservoirs from some carnivore families that are known to have RABV reservoirs, and that some known carnivore reservoirs were predicted to be non-reservoirs. For example, the GBM models identified only two new RABV reservoirs for Mephitidae, both of which had low Shapley scores (i.e. Shapley scores of 0.01 and 0.05 for the pygmy spotted skunk (*Spilogale pygmaea*) and American hog-nosed skunk (*Conepatus leuconotus*), respectively). This suggests that species from this family possibly are less likely to be reservoirs for RABV. Similarly, our conservative GBM model predicted the raccoon (*Procyon lotor*) and the kinkajou (*Potos flavus*) to be non-reservoirs for RABV. One reason for this could be that the number and types of traits included in our GBM models were not sufficient to correctly predict the reservoir status of species that are part of the Procyonidae family. The carnivore models only predicted reservoir status 65–67% of the time (although sensitivity was ~70–76%). Thus, it is possible that our GBM models could be missing an important ecological dimension, suggesting that additional information on hosts that more closely relate to the maintenance of RABV is needed to strengthen future models. Additionally, the difference in the number of species in each family that are currently recognized as RABV reservoirs could also be influencing predictions. For example, in exploratory GBM runs, we found that predictions were sensitive to the composition of the training set, particularly for members of the Procyonidae family. This was likely because fewer than a quarter of known carnivore RABV reservoirs are from the Procyonidae family. This highlights the need for more studies on RABV reservoir status of other members of the Procyonidae family as well as the development of cross-validation approaches that account for phylogenetic structure [68].

Similarly, some of the predicted carnivore RABV reservoirs identified are unlikely to contribute substantially to endemic RABV circulation as they are classified as endangered in the IUCN Red List (e.g., the dhole (*Cuon alpinus*) and the African wild dog (*Lycaon pictus*)). Our GBM models likely predicted these species to be reservoirs for RABV because our reservoir classifications were based solely on species life-history characteristics and did not account for some species occurring in small and fragmented populations that might be unable to maintain RABV. Hence, while identified endangered species are likely not current RABV reservoirs, their life-history characteristics suggest that they have the potential to be. From a conservation standpoint, identifying endangered species as potential reservoirs for RABV reinforces the need to establish surveillance programs for these species so that transmission can more readily be controlled should an outbreak occur.

The geographic clustering of known carnivore reservoirs in Eastern and Southern Africa and North America is probably associated in part with sampling bias. However, examining the geographic distribution of predicted carnivore reservoirs revealed that several predicted carnivore species occur in areas where known reservoir species occur. The conservative model predicted some carnivore reservoirs to occur in southern and central parts of the US and, the liberal model predicted carnivore reservoirs around East Africa and parts of Central and Southern Africa, which for the latter is consistent with previous work on carnivore zoonotic pathogens [14]. As such, while several carnivore reservoirs have been identified in these RABV hotspots, it is possible that several other carnivore species could facilitate RABV maintenance in these regions, and therefore, threaten the effectiveness of ongoing rabies control programs. However, while the predicted reservoirs could contribute to the transmission cycles of existing variants, they could also sustain undiscovered RABV variants.

As in the carnivore analysis, one of the most important traits for being a bat RABV reservoir was litter size, which is consistent with previous work for other types of bat viruses [69]. While litter size can be a proxy for host density for carnivores, it is most often not the case for bats. For example, several bat species that have more than one young per litter tend to be solitary or live in small groups (e.g., the southern yellow bat (*Lasiurus ega*)) while several bat species that have only one young per litter tend to live in large groups (e.g., the Mexican free-tailed bat (*Tadarida brasiliensis*)). Thus, we suspect that the litter size finding is not a reflection of host density in bats. Further, since RABV transmission in bats is more likely frequency than density dependent [70,71], we suspect that a more plausible explanation for the litter size finding is that there is another trait unique to species with more than one young per litter that is driving this association. This highlights a need to explore the importance of other life-history traits at influencing the RABV reservoir status of bats.

We expected phylogenetic grouping to be a primary predictor of RABV reservoirs status for bats since RABV transmission and establishment is more likely to occur between closely than distantly related species [69,72–74]. Despite this, phylogenetic grouping appeared as fifth most important in the conservative model and one of the least important in the liberal model. This finding could be due to data deficiency, or because most of the predicted species were from three of the 21 phylogenetically distinct families (Vespertillionidae, Molossidae, and Phyllostomidae). Further, phylogenetic grouping was likely important at predicting bat RABV reservoir status but did not rank highly, possibly because traits associated with the spatial distribution of species (e.g., species geographic range) were more influential. Likewise, a bat trait that has previously been identified as important for RABV occurrence is diet [75]. Yet, in our models diet ranked as one of the least important predictors of RABV reservoir status for bats. This could be associated with the fact that over 41% of bat species had missing information on their diet status. Thus, more research is needed to determine whether diet is an important predictor of reservoir status for bats.

The fact that the top-ranking traits associated with bats being RABV reservoirs were those associated with the species’ spatial distribution may reflect geographic biases in the tendency for bats to have been reported as RABV reservoirs. For example, latitudinal gradient was one of the top predictors, where bat reservoirs are more prone to occur in North America, which could be a result of there being far greater RABV surveillance in North America than in Central and South America [75–77]. Further, the greater importance of species spatial distribution over life-history characteristics highlights that data on bat ecological and life-history characteristics are alarmingly deficient. For instance, eleven traits in the PanTHERIA database were excluded from our analyses because over 50% of bat species had missing values, and a large proportion of species with missing data occurred in Central and South America. Gathering data on traits that are known to influence RABV transmission and maintenance in bats (e.g., overwintering activity, migration, and roosting behavior; [78]) and focusing efforts on species that have little information would help inform predictive models such as the ones developed here.

The difference in accuracies between the carnivore and bat models is noteworthy. The carnivore models likely had a lower accuracy than the bat models partly because one or several carnivore traits important for RABV maintenance were missing. That said, while the bat models had greater accuracies than the carnivore models, the carnivore findings were generally more insightful than the bat findings because more life-history traits were examined. The high predictive power of the bat models was partly driven by traits that were associated with sampling bias (e.g., location in the Americas). Thus, while both models are useful for identifying traits and potential reservoirs for RABV, they also identify key gaps in both the carnivore and bat datasets. Several additional factors associated with RABV transmission and maintenance should be explored. For example, in addition to traits associated with host density and activity (e.g., population size and roosting behavior), an important factor relates to RABV circulation in species range. The reservoir status of many carnivore and bat species is probably influenced by the number and types of RABV variants circulating in the region, increasing the probability of host shifts. Exploring the importance of such a variable could help tease apart the reservoir status of many species but necessitates that more information on RABV variants be collected and made available.

Our definition of RABV reservoir is a potential limitation of this study. With our definition, species are predicted to be reservoirs across their entire geographic range when in many cases it is populations rather than species that tend to be defined as RABV reservoirs. For example, known reservoirs of the Mephitidae (i.e., the striped skunk (*Mephitis mephitis*) and the eastern spotted skunk (*Spilogale putorius*)) and Procyonidae families (e.g. the raccoon (*Procyon lotor*)) act as reservoirs, but only in certain regions. The Striped skunk, for instance, is considered to be a reservoir for RABV in the southern, central US but not on the eastern coast of the US [34]. Determining which species are likely to be RABV reservoirs across their entire range versus only in certain regions would be an important next step to take. Another potential drawback is our criteria for defining non-reservoirs. We did not account for differences in sampling effort for each species, meaning that our definition of ‘a non-reservoir’ does not make a distinction between ‘evidence that species is not a reservoir’ and ‘data insufficient’. This is a weakness of many similar approaches, suggesting that future work to address this gap is needed. Our online ReservoirFinder database (https://github.com/whit1951/ResevoirFinder) will provide a valuable resource for future RABV reservoir models when new information is available.

Despite these weaknesses, the list of predicted RABV reservoirs identified as part of this study can be used to help target surveillance and control programs. Further, identification of species for which RABV reservoir status was predicted to be uncertain (i.e., Shapley values less than 0.1) is valuable as it provides direction on the types of species for which more research is needed (on both species ecological characteristics and association with genetically distinct virus variants). However, the list of predicted RABV reservoirs should also be considered with a degree of caution for several reasons. Firstly, predictions made are based on the combined effect of the specific traits examined in this study. This means that any addition or removal of traits has the potential to alter the predicted reservoir status of certain species, especially those species that have Shapley values less than 0.1. Similarly, as new information is gathered for missing traits, model predictions will also likely shift. Thus, this study should be viewed as a preliminary step towards identifying current and future RABV reservoirs. In this way, the findings should be used to help focus current and future rabies research and surveillance efforts, but should not replace generalized surveillance. Indeed, some species that the GBM models predicted as non-reservoirs could be reservoirs but traits examined and/or missing data prohibited the GBM models to identify them as RABV reservoirs. In conclusion, by using advances in machine learning, we predicted previously unidentified carnivore and bat reservoirs of RABV that could be targeted in current and future rabies surveillance programs. Further, by investigating the geographic range of known and predicted RABV reservoirs, we provided insight into the locations where RABV in wildlife communities is likely to persist and where future spillover and host shift events are most expected to occur. Using the Shapley value to understand how each trait contributed to the reservoir status of each species was particularly insightful, and we recommend this approach be used to identify additional reservoirs for RABV as more data become available, and for other zoonotic pathogens. Efforts to control rabies in wildlife should aim to prevent RABV host shifts into carnivore and bat species predicted to be RABV reservoirs.

## Supporting information

S1 Table Model performance. (PDF)

S2 Table Carnivore species predicted to be RABV reservoirs based on the liberal criteria. Since there is inherent variation when performing permutations, species with Shapley values close to zero (especially those < 0.1) should be considered with caution.
(PDF)

S3 Table Bat species predicted to be RABV reservoirs based on the liberal criteria. Since there is inherent variation when performing permutations, species with Shapley values close to zero (especially those < 0.1) should be considered with caution.
(PDF)

S1 Fig Principal Coordinates Analysis (PCoA) plot of the patristic distance (pairwise sum of branch lengths between each taxa) for a) carnivores and b) bats. See S2 Fig and the main text for details on the phylogenies used for each group. Names of only a few taxa are provided to aid interpretability.
(TIF)

S2 Fig Species-level carnivore tree illustrating the phylogenetic distribution of RABV reservoirs. Known RABV reservoir species are depicted with red circles (dark red circles are based on the conservative criteria and light red circles on the liberal criteria). Predicted reservoir species are depicted with blue squares (dark blue squares are based on the conservative criteria and light blue squares on the liberal criteria). Colored boxes illustrate the phylogenetic pattern of reservoir status for some of the primary reservoir groups in higher resolution. Red branches and bold text indicate that species may play a role in the maintenance of RABV based on the data or model predictions. Note that not all Mustelidae are shown in the inset. The phylogenetic tree was retrieved from [47]. Silhouettes were downloaded from Phylopic (http://phylopic.org/).
(TIF)

S3 Fig Traits predictive of carnivore RABV reservoir status based on the liberal criteria. **(A)** Trait importance (measured based on model error change after permutation) and **(B)-(F)** partial dependence plots relating reservoir status (the log-odds scale of being a reservoir for RABV) with the five most predictive carnivore traits based on the liberal criteria. In panel **(A)**, PCoA1-3 refers to principal coordinates 1 through 3 of species phylogenetic relatedness. In panels **(B)-(D)** and **(F)**, the red line represents the mean prediction across all species. The grey lines are the Individual Conditional Expectation (ICE) curves, which illustrate the predictive change in each species being a reservoir for RABV as each feature changes. The tick marks along the x-axis represent the deciles of each trait values included in model training. The median age at sexual maturity was ~2 years and the median litter size was 2.35.
(TIF)

S4 Fig Traits predictive of bat RABV reservoir status based on the liberal criteria. **(A)** Trait importance (measured based on model error change after permutation) and **(B)-(E)** partial dependence plots relating reservoir status (the log-odds scale of being a reservoir for RABV) with the four most predictive bat traits based on the liberal criteria. In panel **(A)**, PCoA1-4 refers to principal coordinates 1 through 4 of species phylogenetic relatedness. In panels **(B), (D)**, and **(E)**, the red line represents the mean prediction across all species. The grey lines are the ICE curves, which illustrate the predictive change in each species being a reservoir for RABV as each feature changes. The tick marks along the x-axis represent the deciles of each trait values included in model training.
(TIF)

## References

[1] Haydon DT, Cleaveland S, Taylor LH, Laurenson MK. Identifying reservoirs of infection: A conceptual and practical challenge. Emerg Infect Dis. 2002;8: 1468–1473. 10.3201/eid0812.010317 12498665 PMC2738515

[2] Viana M, Cleaveland S, Matthiopoulos J, Halliday J, Packer C, Craft ME, et al Dynamics of a morbillivirus at the domestic–wildlife interface: Canine distemper virus in domestic dogs and lions. Proc Natl Acad Sci. 2015;112: 1464–1469. 10.1073/pnas.1411623112 25605919 PMC4321234

[3] Streicker DG, Fenton A, Pedersen AB. Differential sources of host species heterogeneity influence the transmission and control of multihost parasites. Ecol Lett. 2013;16: 975–984. 10.1111/ele.12122 23714379 PMC4282463

[4] Babayan SA, Orton RJ, Streicker DG. Predicting reservoir hosts and arthropod vectors from evolutionary signatures in RNA virus genomes. Science. 2018;362: 577–580. 10.1126/science.aap9072 30385576 PMC6536379

[5] Lloyd-Smith JO, George D, Pepin KM, Pitzer VE, Pulliam JRC, Dobson AP, et al Epidemic Dynamics at the Human-Animal Interface. Science. 2009;326: 1362–1367. 10.1126/science.1177345 19965751 PMC3891603

[6] Plowright RK, Parrish CR, McCallum H, Hudson PJ, Ko AI, Graham AL, et al Pathways to zoonotic spillover. Nat Rev Microbiol. 2017;15: 502–510. 10.1038/nrmicro.2017.45 28555073 PMC5791534

[7] Vosloo W, Thompson PN, Botha B, Bengis RG, Thomson GR. Longitudinal study to investigate the role of impala (Aepyceros melampus) in foot-and-mouth disease maintenance in the Kruger National Park, South Africa. Transbound Emerg Dis. 2009;56: 18–30. 10.1111/j.1865-1682.2008.01059.x 19200295

[8] Diffenbaugh NS, Field CB. Changes in ecologically critical terrestrial climate conditions. Science. 2013;341: 486–492. 10.1126/science.1237123 23908225

[9] Sunday JM, Bates AE, Dulvy NK. Thermal tolerance and the global redistribution of animals. Nat Clim Chang. 2012;2: 686–690. 10.1038/NCLIMATE1539

[10] Williams JE, Blois JL. Range shifts in response to past and future climate change: Can climate velocities and species’ dispersal capabilities explain variation in mammalian range shifts? J Biogeogr. 2018;45: 2175–2189. 10.1111/jbi.13395

[11] Luis AD, Hayman DTS, O’Shea TJ, Cryan PM, Gilbert AT, Pulliam JRC, et al A comparison of bats and rodents as reservoirs of zoonotic viruses: are bats special? Proc R Soc B Biol Sci. 2013;280: 20122753 10.1098/rspb.2012.2753 23378666 PMC3574368

[12] Mcart SH, Koch H, Irwin RE, Adler LS. Arranging the bouquet of disease: Floral traits and the transmission of plant and animal pathogens. Ecol Lett. 2014;17: 624–636. 10.1111/ele.12257 24528408

[13] Estrada-Peña A, Ostfeld RS, Peterson AT, Poulin R, De La Fuente J. Effects of environmental change on zoonotic disease risk: An ecological primer. Trends Parasitol. 2014;30: 205–214. 10.1016/j.pt.2014.02.003 24636356

[14] Olival KJ, Hosseini PR, Zambrana-Torrelio C, Ross N, Bogich TL, Daszak P. Host and viral traits predict zoonotic spillover from mammals. Nature. 2017;546: 646–650. 10.1038/nature22975 28636590 PMC5570460

[15] Han BA, Schmidt JP, Bowden SE, Drake JM. Rodent reservoirs of future zoonotic diseases. Proc Natl Acad Sci. 2015;112: 7039–7044. 10.1073/pnas.1501598112 26038558 PMC4460448

[16] Plourde BT, Burgess TL, Eskew EA, Roth TM, Stephenson N, Foley JE. Are disease reservoirs special? Taxonomic and life history characteristics. PLoS One. 2017;12: 1–23. 10.1371/journal.pone.0180716 28704402 PMC5509157

[17] Johnson PTJ, Rohr JR, Hoverman JT, Kellermanns E, Bowerman J, Lunde KB. Living fast and dying of infection: host life history drives interspecific variation in infection and disease risk. Ecol Lett. 2012;15: 235–242. 10.1111/j.1461-0248.2011.01730.x 22221837

[18] Huang ZYX, de Boer WF, van Langevelde F, Olson V, Blackburn TM, Prins HHT. Species’ life-history traits explain interspecific variation in reservoir competence: A possible mechanism underlying the dilution effect. PLoS One. 2013;8: 1–6. 10.1371/journal.pone.0054341 23365661 PMC3554779

[19] Pandit PS, Doyle MM, Smart KM, Young CCW, Drape GW, Johnson CK. Predicting wildlife reservoirs and global vulnerability to zoonotic Flaviviruses. Nat Commun. 2018;9: 1–10. 30575757 10.1038/s41467-018-07896-2 PMC6303316

[20] Ostfeld RS, Levi T, Jolles AE, Martin LB, Hosseini PR, Keesing F. Life history and demographic drivers of reservoir competence for three tick-borne zoonotic pathogens. PLoS One. 2014;9 10.1371/journal.pone.0107387 25232722 PMC4169396

[21] Hampson K, Coudeville L, Lembo T, Sambo M, Kieffer A, Attlan M, et al Estimating the global burden of endemic canine rabies. PLoS Negl Trop Dis. 2015;9: e0003709 10.1371/journal.pntd.0003709 25881058 PMC4400070

[22] WHO. Expert Consultation on Rabies: Second Report. Tech. Rep. Ser. Geneva; 2013. 24069724

[23] Mollentze N, Biek R, Streicker DG. The role of viral evolution in rabies host shifts and emergence. Curr Opin Virol. 2014;8: 68–72. 10.1016/j.coviro.2014.07.004 25064563 PMC4199325

[24] Chernet B, Nejash A. Review of rabies preventions and control. World Appl Sci J. 2016;34: 1422–1429. 10.5829/idosi.wasj.2016.1422.1429

[25] Cleaveland S, Dye C. Maintenance of a microparasite infecting several host species: Rabies in the Serengeti. Parasitology. 1995;111: S33–S47. 10.1017/s0031182000075806 8632923

[26] Bourhy H, Dautry-Varsat A, Hotez PJ, Rô Me Salomon J. Rabies, still neglected after 125 years of vaccination. PLoS Negl Trop Dis. 2010;4 10.1371/journal.pntd.0000839 21152052 PMC2994912

[27] Cleaveland S, Hampson K. Rabies elimination research: Juxtaposing optimism, pragmatism and realism. Proc R Soc B Biol Sci. 2017;284: 20171880 10.1098/rspb.2017.1880 29263285 PMC5745407

[28] Nel LH, Sabeta CT, von Teichman B, Jaftha JB, Rupprecht CE, Bingham J. Mongoose rabies in southern Africa: A re-evaluation based on molecular epidemiology. Virus Res. 2005;109: 165–173. 10.1016/j.virusres.2004.12.003 15763147

[29] Zulu GC, Sabeta CT, Nel LH. Molecular epidemiology of rabies: Focus on domestic dogs (*Canis familiaris*) and black-backed jackals (*Canis mesomelas*) from northern South Africa. Virus Res. 2009;140: 71–78. 10.1016/j.virusres.2008.11.004 19061924

[30] Cordeiro R de A, Duarte NFH, Rolim BN, Soares Júnior FA, Franco ICF, Ferrer LL, et al The importance of wild canids in the epidemiology of rabies in northeast Brazil: A retrospective study. Zoonoses Public Health. 2016;63: 486–493. 10.1111/zph.12253 26815766

[31] Vercauteren KC, Ellis C, Chipman R, Deliberto TJ, Shwiff SA. Rabies in North America: A model of the One Health approach. Proceedings of the 14th WDM Conference. 2012. pp. 56–63.

[32] Belotto A, Leanes LF, Schneider MC, Tamayo H, Correa E. Overview of rabies in the Americas. Virus Res. 2005;111: 5–12. 10.1016/j.virusres.2005.03.006 15896398

[33] Velasco-Villa A, Reeder SA, Orciari LA, Yager PA, Franka R, Blanton JD, et al Enzootic rabies elimination from dogs and reemergence in wild terrestrial carnivores, United States. Emerg Infect Dis. 2008;14: 1849 10.3201/eid1412.080876 19046506 PMC2634643

[34] Wallace RM, Gilbert A, Slate D, Chipman R, Singh A, Wedd C, et al Right place, wrong species: A 20-year review of rabies virus cross species transmission among terrestrial mammals in the United States. PLoS One. 2014;9 10.1371/journal.pone.0107539 25295750 PMC4189788

[35] Blanton JD, Dyer J, McBrayer J, Rupprecht CE. Rabies surveillance in the United States during 2011. J Am Vet Med Assoc. 2012;241 10.2460/javma.241.6.712 22947154 PMC5120402

[36] Birhane MG, Cleaton JM, Monroe BP, Wadhwa A, Orciari LA, Yager P, et al Rabies surveillance in the United States during 2015. J Am Vet Med Assoc. 2017;250: 1117–1130. 10.2460/javma.250.10.1117 28467751

[37] Blanton JD, Palmer D, Dyer J, Rupprecht CE. Rabies surveillance in the United States during 2010. J Am Vet Med Assoc. 2011;239: 712–722. 10.2460/javma.239.6.773 21916759 PMC5120392

[38] Kuzmin I V., Shi M, Orciari LA, Yager PA, Velasco-Villa A, Kuzmina NA, et al Molecular inferences suggest multiple host shifts of rabies viruses from bats to mesocarnivores in Arizona during 2001–2009. PLoS Pathog. 2012;8: e1002786 10.1371/journal.ppat.1002786 22737076 PMC3380930

[39] Ding NZ, Xu DS, Sun YY, He H Bin, He CQ. A permanent host shift of rabies virus from Chiroptera to Carnivora associated with recombination. Sci Rep. 2017;7: 1–9. 28325933 10.1038/s41598-017-00395-2 PMC5428239

[40] Velasco-Villa A, Mauldin MR, Shi M, Escobar LE, Gallardo-Romero NF, Damon I, et al The history of rabies in the Western Hemisphere. Antiviral Res. 2017;146: 221–232. 10.1016/j.antiviral.2017.03.013 28365457 PMC5620125

[41] Gilbert A T. Rabies virus vectors and reservoir species. Rev Sci Tech. 2018;37: 371–384. 10.20506/rst.37.2.2808 30747141

[42] Rupprecht CE, Hanlon CA, Hemachudha T. Rabies re-examined. Lancet Infect Dis. 2002;2: 327–343. 10.1016/s1473-3099(02)00287-6 12144896

[43] Rupprecht C, Kuzmin I, Meslin F. Lyssaviruses and rabies: Current conundrums, concerns, contradictions and controversies. F1000Research. 2017;6 10.12688/f1000research.10416.1 28299201 PMC5325067

[44] Fisher CR, Streicker DG, Schnell MJ. The spread and evolution of rabies virus: conquering new frontiers. Nat Rev Microbiol. 2018;16 4: 241 10.1038/nrmicro.2018.11 29479072 PMC6899062

[45] Troupin C, Dacheux L, Tanguy M, Sabeta C, Blanc H, Bouchier C, et al Large-scale phylogenomic analysis reveals the complex evolutionary history of rabies virus in multiple carnivore hosts. PLoS Pathog. 2016;12 10.1371/journal.ppat.1006041 27977811 PMC5158080

[46] Jones KE, Bielby J, Cardillo M, Fritz SA, O’dell J, David C, et al PanTHERIA: A species-level database of life history, ecology, and geography of extant and recently extinct mammals. Ecology. 2009;90: 2648 10.1890/08-1494.1

[47] Nyakatura K, Bininda-Emonds OR. Updating the evolutionary history of Carnivora (Mammalia): a new species-level supertree complete with divergence time estimates. BMC Biol. 2012;10: 12 10.1186/1741-7007-10-12 22369503 PMC3307490

[48] Agnarsson I, Zambrana-Torrelio CM, Flores-Saldana NP, May-Collado LJ. A time-calibrated species-level phylogeny of bats (Chiroptera, Mammalia). PLoS Curr. 2011;3 10.1371/currents.RRN1212 21327164 PMC3038382

[49] Felsenstein J. Phylogenies and the Comparative Method. Am Nat. 1985;125: 1–15. 10.1086/284325

[50] R Development Core Team. R: A language and environment for statistical computing. Viana, Austria; 2020.

[51] Greenwell B, Boehmke B, Cunningham J, Developers G. Package “gbm” Generalized Boosted Regression Models. 2020.

[52] Kuhn M, Wing J, Weston S, Williams A, Keefer C, Engelhardt A, et al Package ‘ caret ‘ Classification and Regression Training. 2020.

[53] Friedman JH. Greedy function approximation: A gradient boosting machine. Ann Stat. 2001;29: 1189–1232.

[54] Elith J, Leathwick JR, Hastie T. A working guide to boosted regression trees. J Anim Ecol. 2008;77: 802–813. 10.1111/j.1365-2656.2008.01390.x 18397250

[55] Fountain-Jones NM, Machado G, Carver S, Packer C, Recamonde-Mendoza M, Craft ME. How to make more from exposure data? An integrated machine learning pipeline to predict pathogen exposure. J Anim Ecol. 2019;88: 1447–1461. 10.1111/1365-2656.13076 31330063

[56] Kuhn M. Building predictive models in R using the caret package. J Stat Softw. 2008;28: 1–26.27774042

[57] Molnar C, Casalicchio G, Bischl B. iml: An R package for interpretable machine learning. J Open Source Softw. 2018;3: 786 10.21105/joss.00786

[58] Fisher A, Rudin C, Dominici F. All models are wrong but many are useful: Variable Importance for black-box, proprietary, or misspecified prediction models, using model class reliance. J Mach Learn Res. 2018; 237–246. PMC8323609 34335110

[59] Greenwell BM. pdp: An R package for constructing partial dependence plots. R J. 2017;9: 421–436.

[60] Goldstein A, Kapelner A, Bleich J, Pitkin E. Peeking inside the black box: Visualizing statistical learning with plots of individual conditional expectation. J Comput Graph Stat. 2015;24: 44–65. 10.1080/10618600.2014.907095

[61] Shapley LS. Introduction to the Shapley value Contributions to the Theory of Games. Cambridge: Cambridge University Press; 1953 pp. 307–317.

[62] Bivand R, Keitt T, Rowlingson B. rgdal: Bindings for the “Geospatial” Data Abstraction Library R package version 1.4–8. 2019.

[63] Lamigueiro OP, Hijmans R, Lamigueiro MOP. rasterVis R package version 0.47. 2019 https://oscarperpinan.github.io/rastervis/

[64] Han BA, Park AW, Jolles AE, Altizer S. Infectious disease transmission and behavioural allometry in wild mammals. J Anim Ecol. 2015;84: 637–646. 10.1111/1365-2656.12336 25631200

[65] Anderson RM, Jackson HC, May RM, Smith AM. Population dynamics of fox rabies in Europe. Nature. 1981;289: 765 10.1038/289765a0 7464941

[66] Hampson K, Dushoff J, Cleaveland S, Haydon DT, Kaare M, Packer C, et al Transmission dynamics and prospects for the elimination of canine rabies. PLoS Biol. 2009;7: 462–471. 10.1371/journal.pbio.1000053 19278295 PMC2653555

[67] Morters MK, Restif O, Hampson K, Cleaveland S, Wood JLN, Conlan AJK. Evidence-based control of canine rabies: a critical review of population density. J Anim Ecol. 2013;82: 6–14. 10.1111/j.1365-2656.2012.02033.x 23004351 PMC3579231

[68] Roberts DR, Bahn V, Ciuti S, Boyce MS, Elith J, Guillera-Arroita G, et al Cross-validation strategies for data with temporal, spatial, hierarchical, or phylogenetic structure. Ecography. 2017;40: 913–929. 10.1111/ecog.02881

[69] Guy C, Thiagavel J, Mideo N, Ratcliffe JM. Phylogeny matters: Revisiting “a comparison of bats and rodents as reservoirs of zoonotic viruses.” R Soc Open Sci. 2019;6: 181182 10.1098/rsos.181182 30891262 PMC6408376

[70] Streicker DG, Recuenco S, Valderrama W, Benavides JG, Vargas I, Pacheco V, et al Ecological and anthropogenic drivers of rabies exposure in vampire bats: implications for transmission and control. Proc R Soc B Biol Sci. 2012;279: 3384–3392. 10.1098/rspb.2012.0538 22696521 PMC3396893

[71] Blackwood JC, Streicker DG, Altizer S, Rohani P. Resolving the roles of immunity, pathogenesis, and immigration for rabies persistence in vampire bats. Proc Natl Acad Sci. 2013;110: 20837–20842. 10.1073/pnas.1308817110 24297874 PMC3870737

[72] Streicker DG, Turmelle AS, Vonhof MJ, Kuzmin I V, McCracken GF, Rupprecht CE. Host phylogeny constrains cross-species emergence and establishment of rabies virus in bats. Science. 2010;329: 676–9. 10.1126/science.1188836 20689015

[73] Faria NR, Suchard MA, Rambaut A, Streicker DG, Lemey P. Simultaneously reconstructing viral cross-species transmission history and identifying the underlying constraints. Philos Trans R Soc B Biol Sci. 2013;368: 20120196 10.1098/rstb.2012.0196 23382420 PMC3678322

[74] Luis AD, O’Shea TJ, Hayman DTS, Wood JLN, Cunningham AA, Gilbert AT, et al Network analysis of host-virus communities in bats and rodents reveals determinants of cross-species transmission. Ecol Lett. 2015;18: 1153–1162. 10.1111/ele.12491 26299267 PMC5014217

[75] Escobar. Bat-borne rabies in Latin America. Rev Inst Med Trop Sao Paulo. 2015;57: 63–72. 10.1590/S0036-46652015000100009 25651328 PMC4325525

[76] Velasco-Villa A, Orciari LA, Juárez-Islas V, Gómez-Sierra M, Padilla-Medina I, Flisser A, et al Molecular diversity of rabies viruses associated with bats in Mexico and other countries of the Americas. J Clin Microbiol. 2006;44: 1697–1710. 10.1128/JCM.44.5.1697-1710.2006 16672396 PMC1479161

[77] Kuzmin I V, Bozick B, Guagliardo SA, Kunkel R, Shak JR, Tong S, et al Bats, emerging infectious diseases, and the rabies paradigm revisited. Emerg Health Threats J. 2011;4: 7159 10.3402/ehtj.v4i0.7159 24149032 PMC3168224

[78] Streicker DG, Lemey P, Velasco-Villa A, Rupprecht CE. Rates of viral evolution are linked to host geography in bat rabies. PLoS Pathog. 2012;8: e1002720 10.1371/journal.ppat.1002720 22615575 PMC3355098

