# Supplementary material for: Using host traits to predict reservoir host species of rabies virus
Source: PLoS Negl Trop Dis. 2020 Dec 8;14(12):e0008940. doi: 10.1371/journal.pntd.0008940 (PMC7748407; doi:10.1371/journal.pntd.0008940)
Supplement: S1 Table — (PDF) [file pntd.0008940.s001.pdf]

S1 Table. Model performance

Model	Accuracy	Specificity	Sensitivity
Carnivore - conservative model	67.16	67.07	75.79
Carnivore - liberal model	65.89	65.84	70.0
Bat - conservative model	82.59	82.57	83.75
Bat - liberal model	82.41	82.32	87.58
