# Supplementary material for: Using host traits to predict reservoir host species of rabies virus
Source: PLoS Negl Trop Dis. 2020 Dec 8;14(12):e0008940. doi: 10.1371/journal.pntd.0008940 (PMC7748407; doi:10.1371/journal.pntd.0008940)
Supplement: S2 Table — Since there is inherent variation when performing permutations, species with Shapley values close to zero (especially those < 0.1) should be considered with caution. (PDF) [file pntd.0008940.s002.pdf]

S2 Table. Carnivore species predicted to be RABV reservoirs based on the liberal criteria. Since there is inherent variation when performing permutations, species with Shapley values close to zero (especially those < 0.1) should be considered with caution.

**Species**	**Family**	**Shapley value**
Fennec fox (*Vulpes zerda*)	Canidae	0.30
Swift fox (*Vulpes velox*)	Canidae	0.28
Culpeo (*Lycalopex culpaeus*)	Canidae	0.24
African civet (*Civettictis civetta*)	Viverridae	0.14
Long-tailed weasel (*Mustela frenata*)	Mustelidae	0.22
Cape fox (*Vulpes chama*)	Canidae	0.21
Island fox (*Urocyon littoralis*)	Canidae	0.2
Ring-tailed cat (*Bassariscus astutus*)	Procyonidae	0.2
Steppe polecat (*Mustela eversmanii*)	Mustelidae	0.2
Dhole (*Cuon alpinus*)	Canidae	0.43
Bush dog (*Speothos venaticus*)	Canidae	0.18
Pampas fox (*Lycalopex gymnocercus*)	Canidae	0.17
Common kusimanse (*Crossarchus obscurus*)	Herpestidae	0.15
Maned wolf (*Chrysocyon brachyurus*)	Canidae	0.14
Stoat (*Mustela erminea*)	Mustelidae	0.14
Indian grey mongoose (*Herpestes edwardsi*)	Herpestidae	0.12
Least weasel (*Mustela nivalis*)	Mustelidae	0.12
Striped hyena (*Hyaena hyaena*)	Hyaenidae	0.12
Kit fox (*Vulpes macrotis*)	Canidae	0.11
African wild dog (*Lycaon pictus*)	Canidae	0.11
Tibetan sand fox (*Vulpes ferrilata*)	Canidae	0.1
Banded mongoose (*Mungos mungo*)	Herpestidae	0.09
Striped polecat (*Ictonyx striatus*)	Mustelidae	0.09
Wildcat (*Felis silvestris*)	Felidae	0.08
Honey badger (*Mellivora capensis*)	Mustelidae	0.07
Egyptian mongoose (*Herpestes ichneumon*)	Herpestidae	0.07
Common genet (*Genetta genetta*)	Viverridae	0.07
Pygmy spotted skunk (*Spilogale pygmaea*)	Mephitidae	0.06
Bengal fox (*Vulpes bengalensis*)	Canidae	0.06
Brown bear (*Ursus arctos*)	Ursidae	0.05
African clawless otter (*Aonyx capensis*)	Mustelidae	0.05
Pale fox (*Vulpes pallida*)	Canidae	0.04
American hog-nosed skunk (*Conepatus leuconotus*)	Mephitidae	0.04
European polecat (*Mustela putorius*)	Mustelidae	0.03
Brown palm civet (*Paradoxurus jerdoni*)	Viverridae	0.03
Blandford’s fox (*Vulpes cana*)	Canidae	0.03
Malabar large-spotted civet (*Viverra civettina*)	Viverridae	0.03
Crab-eating raccoon (*Procyon cancrivorus*)	Procyonidae	0.02
American badger (*Taxidea taxus*)	Mustelidae	0.01
