# Supplementary material for: Using host traits to predict reservoir host species of rabies virus
Source: PLoS Negl Trop Dis. 2020 Dec 8;14(12):e0008940. doi: 10.1371/journal.pntd.0008940 (PMC7748407; doi:10.1371/journal.pntd.0008940)
Supplement: S3 Table — Since there is inherent variation when performing permutations, species with Shapley values close to zero (especially those < 0.1) should be considered with caution. (PDF) [file pntd.0008940.s003.pdf]

S3 Table. Bat species predicted to be RABV reservoirs based on the liberal criteria. Since there is inherent variation when performing permutations, species with Shapley values close to zero (especially those < 0.1) should be considered with caution.

Species	Family	Shapley value
Long-legged myotis (*Myotis volans*)	Vespertilionidae	0.38
Riparian myotis (*Myotis riparius*)	Vespertilionidae	0.38
Western mastiff bat (*Eumops perotis*)	Molossidae	0.36
Big free-tailed bat (*Nyctinomops macrotis*)	Molossidae	0.36
Spotted bat (*Euderma maculatum*)	Vespertilionidae	0.36
Dwarf bonneted bat (*Eumops bonariensis*)	Molossidae	0.33
Little yellow-shouldered bat (*Sturnira lilium*)	Phyllostomidae	0.32
Armenian whiskered bat (*Myotis hajastanicus*)	Vespertilionidae	0.31
Dark-nosed small-footed myotis (*Myotis melanorhinus*)	Vespertilionidae	0.29
Greater bulldog bat (*Noctilio leporinus*)	Noctilionidae	0.29
Pallas's long-tongued bat (*Glossophaga soricina*)	Phyllostomidae	0.28
Indiana bat (*Myotis sodalis*)	Vespertilionidae	0.43
Eastern small-footed myotis (*Myotis leibii*)	Vespertilionidae	0.24
Northern long-eared myotis (*Myotis septentrionalis*)	Vespertilionidae	0.24
Gray bat (*Myotis grisescens*)	Vespertilionidae	0.22
Rafinesque's big-eared bat (*Corynorhinus rafinesquii*)	Vespertilionidae	0.12
Yellowish myotis (*Myotis levis*)	Vespertilionidae	0.11
Southwestern myotis (*Myotis auriculus*)	Vespertilionidae	0.1
Allen's big-eared bat (*Idionycteris phyllotis*)	Vespertilionidae	0.09
White-throated round-eared bat (*Lophostoma silvicolum*)	Phyllostomidae	0.09
Atacama myotis (*Myotis atacamensis*)	Vespertilionidae	0.09
California leaf-nosed bat (*Macrotus californicus*)	Phyllostomidae	0.07
Southern myotis (*Myotis aelleni*)	Vespertilionidae	0.06
Southern big-eared brown bat (*Histiotus magellanicus*)	Vespertilionidae	0.06
White-winged vampire bat (*Diaemus youngi*)	Phyllostomidae	0.04
Little yellow bat (*Rhogeessa parvula*)	Vespertilionidae	0.03
Sinaloan mastiff bat (*Molossus sinaloae*)	Molossidae	0.03
Thomas's big-eared brown bat (*Histiotus laephotis*)	Vespertilionidae	0.02
Black-winged little yellow bat (*Rhogeessa tumida*)	Vespertilionidae	0.02
Handley's tailless bat (*Anoura cultrata*)	Phyllostomidae	0.02
Myotis vivesi (*Myotis vivesi*)	Vespertilionidae	0.02
Long-legged bat (*Macrophyllum macrophyllum*)	Phyllostomidae	0.01
Guianan spear-nosed bat (*Phyllostomus latifolius*)	Phyllostomidae	0.01
Pocketed free-tailed bat (*Nyctinomops femorosaccus*)	Molossidae	0.01
