# Supplementary figures and images for: Using host traits to predict reservoir host species of rabies virus
Source: PLoS Negl Trop Dis. 2020 Dec 8;14(12):e0008940. doi: 10.1371/journal.pntd.0008940 (PMC7748407; doi:10.1371/journal.pntd.0008940)

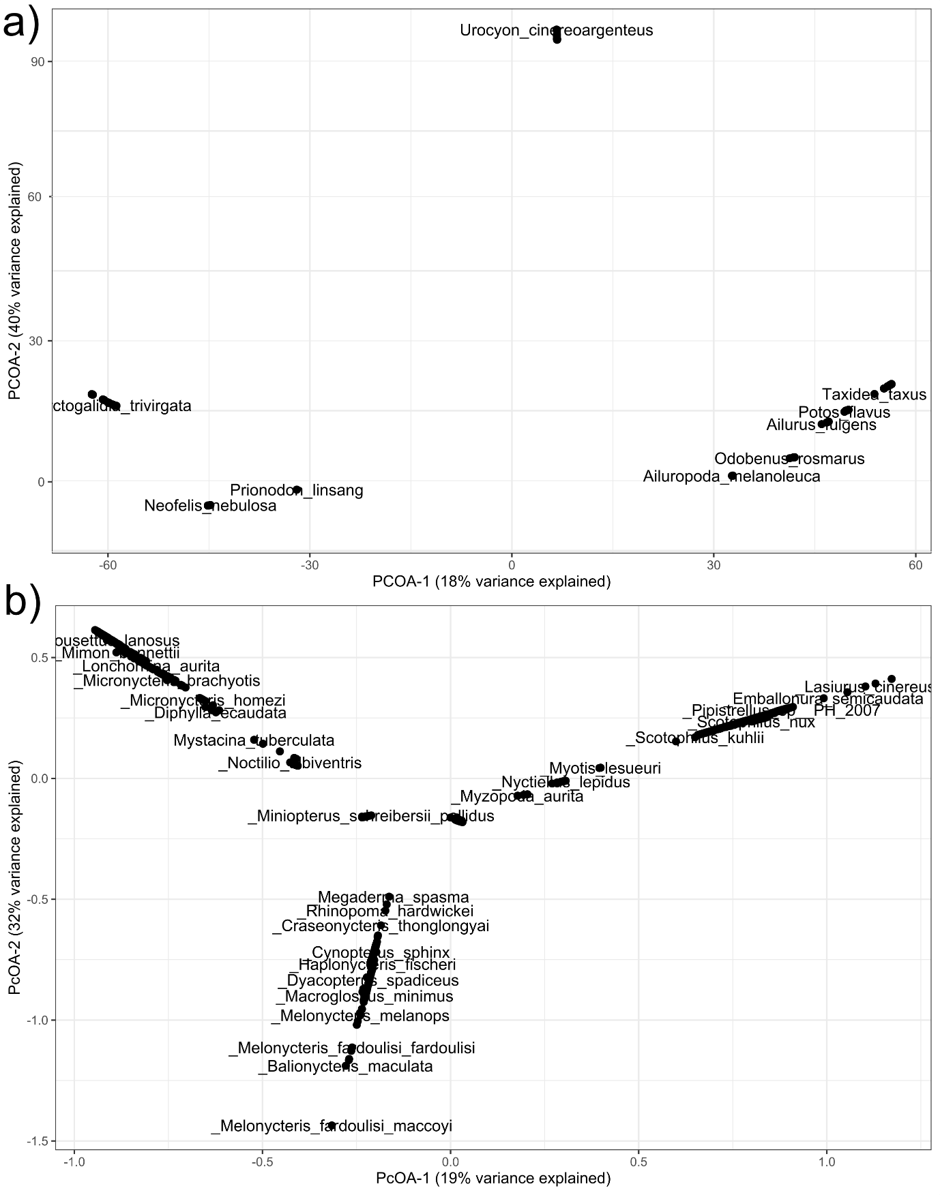

Supplement: S1 Fig — See S2 Fig and the main text for details on the phylogenies used for each group. Names of only a few taxa are provided to aid interpretability. (TIF) [file pntd.0008940.s004.tif]

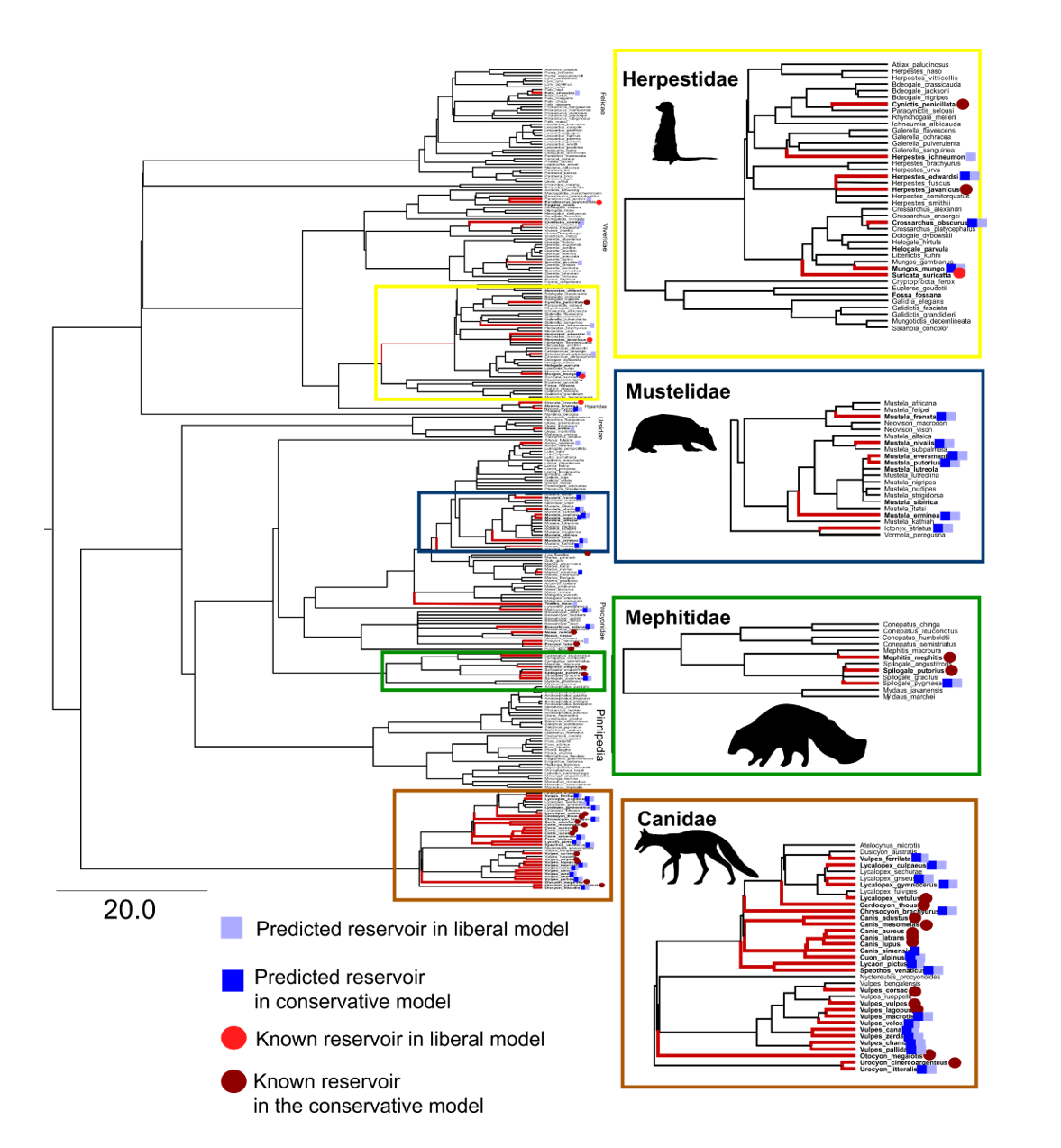

Supplement: S2 Fig — Known RABV reservoir species are depicted with red circles (dark red circles are based on the conservative criteria and light red circles on the liberal criteria). Predicted reservoir species are depicted with blue squares (dark blue squares are based on the conservative criteria and light blue squares on the liberal criteria). Colored boxes illustrate the phylogenetic pattern of reservoir status for some of the primary reservoir groups in higher resolution. Red branches and bold text indicate that species may play a role in the maintenance of RABV based on the data or model predictions. Note that not all Mustelidae are shown in the inset. The phylogenetic tree was retrieved from [47]. Silhouettes were downloaded from Phylopic (http://phylopic.org/). (TIF) [file pntd.0008940.s005.tif]

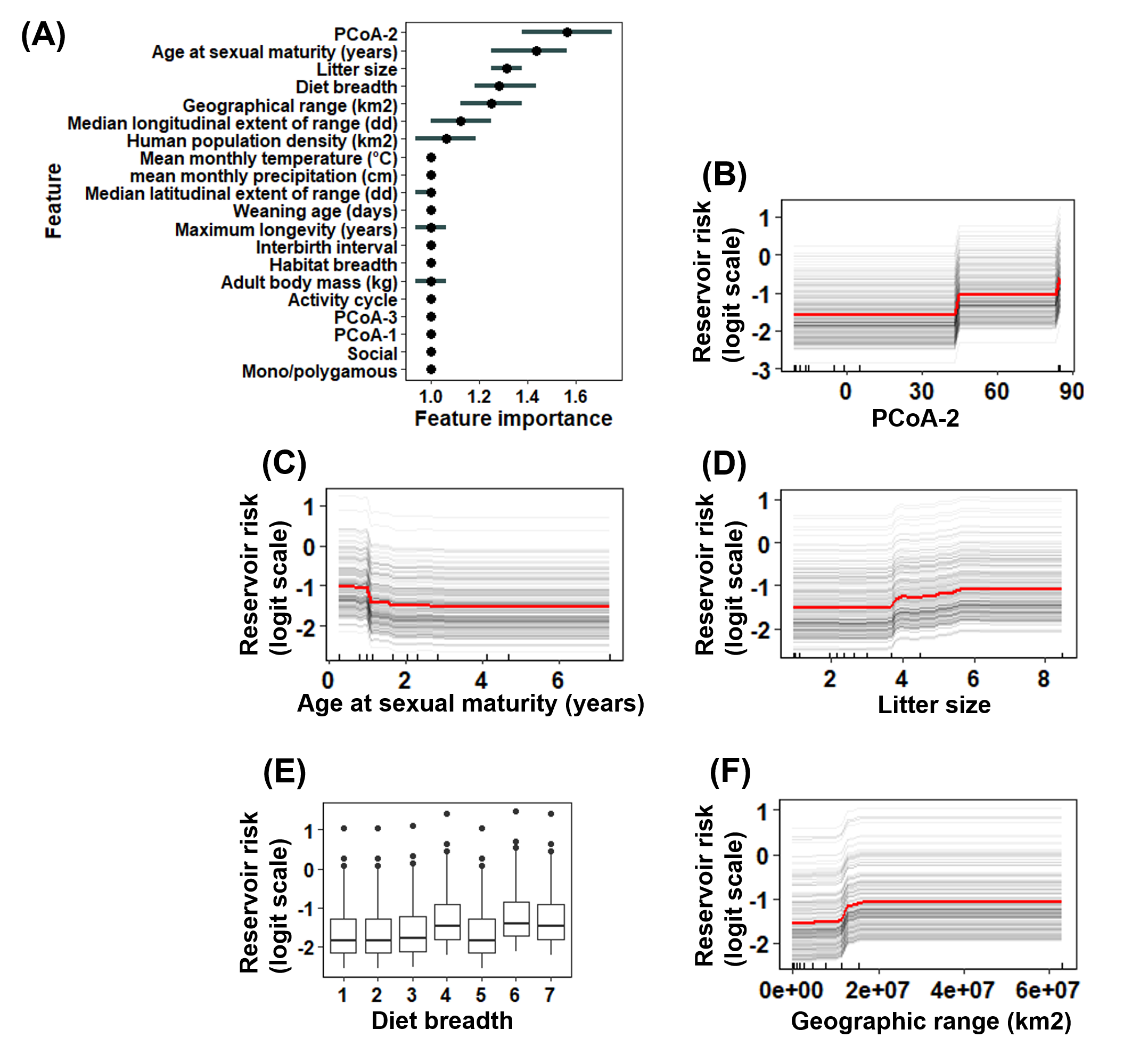

Supplement: S3 Fig — (A) Trait importance (measured based on model error change after permutation) and (B)-(F) partial dependence plots relating reservoir status (the log-odds scale of being a reservoir for RABV) with the five most predictive carnivore traits based on the liberal criteria. In panel (A), PCoA1-3 refers to principal coordinates 1 through 3 of species phylogenetic relatedness. In panels (B)-(D) and (F), the red line represents the mean prediction across all species. The grey lines are the Individual Conditional Expectation (ICE) curves, which illustrate the predictive change in each species being a reservoir for RABV as each feature changes. The tick marks along the x-axis represent the deciles of each trait values included in model training. The median age at sexual maturity was ~2 years and the median litter size was 2.35. (TIF) [file pntd.0008940.s006.tif]

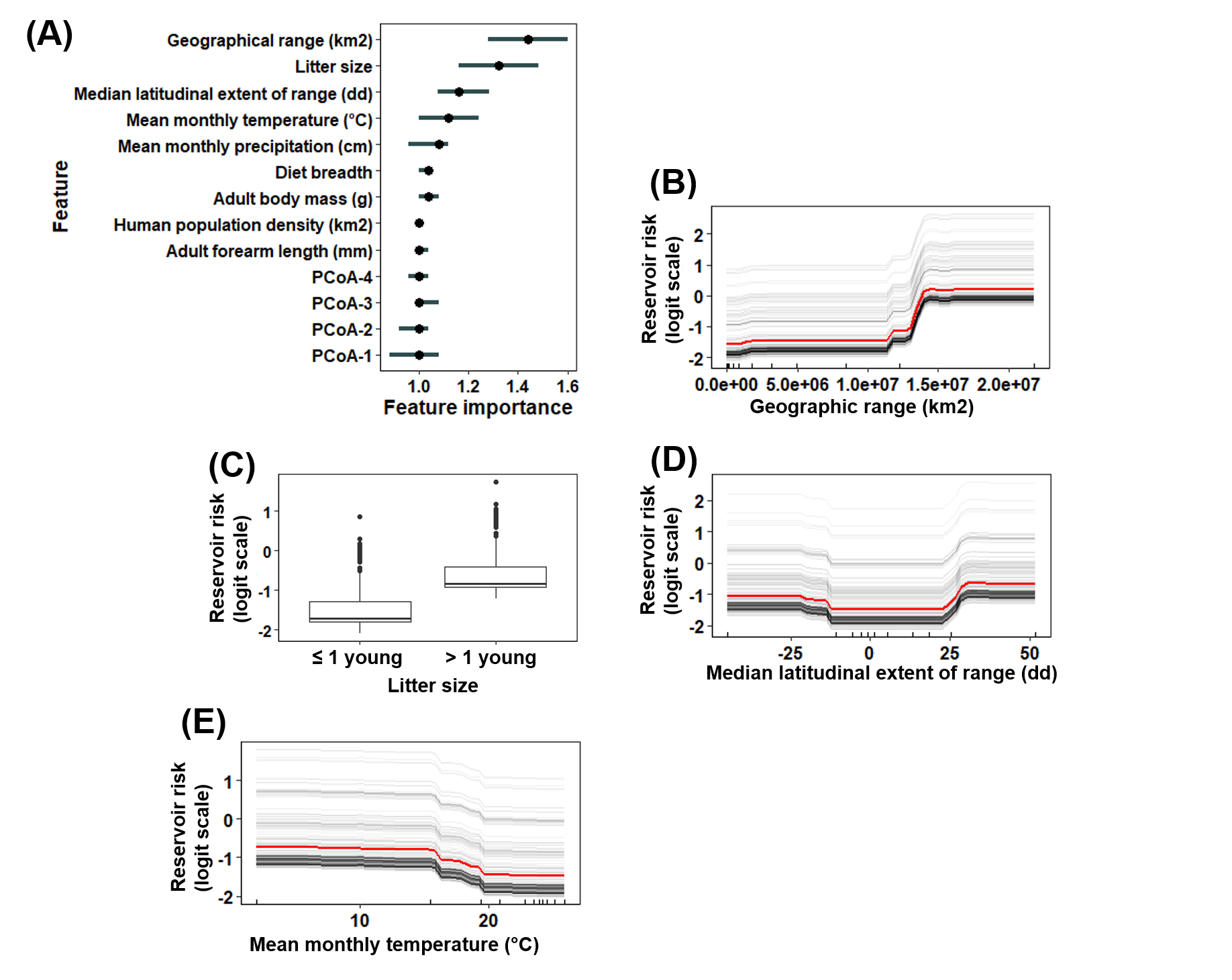

Supplement: S4 Fig — (A) Trait importance (measured based on model error change after permutation) and (B)-(E) partial dependence plots relating reservoir status (the log-odds scale of being a reservoir for RABV) with the four most predictive bat traits based on the liberal criteria. In panel (A), PCoA1-4 refers to principal coordinates 1 through 4 of species phylogenetic relatedness. In panels (B), (D), and (E), the red line represents the mean prediction across all species. The grey lines are the ICE curves, which illustrate the predictive change in each species being a reservoir for RABV as each feature changes. The tick marks along the x-axis represent the deciles of each trait values included in model training. (TIF) [file pntd.0008940.s007.tif]
